# TIM-3 increases the abundance of type-2 dendritic cells during *Leishmania donovani* infection by enhancing IL-10 production via STAT3

**DOI:** 10.1038/s41419-023-05848-3

**Published:** 2023-05-18

**Authors:** Manish Mishra, Manisha Yadav, Sandeep Kumar, Raj Kumar, Pradip Sen

**Affiliations:** 1 Division of Cell Biology and Immunology, Council of Scientific and Industrial Research-Institute of Microbial Technology, Chandigarh, India; 2 Academy of Scientific and Innovative Research (AcSIR), Ghaziabad, India; 3 Present Address: Department of Pathology, Yale School of Medicine, New Haven, CT USA

**Keywords:** Parasitic infection, Infection

## Abstract

The outcome of the disease visceral leishmaniasis (VL), caused by *Leishmania donovani* (LD), largely relies on the relative dominance of host-protective type-1 T helper (Th1) cell response versus disease-promoting type-2 T helper (Th2) cell response. The Th1 and Th2 responses, in turn, are believed to be elicited by type-1 conventional dendritic cells (cDC1) and type-2 conventional DCs (cDC2), respectively. However, it is still unknown which DC subtype (cDC1 or cDC2) predominates during chronic LD infection and the molecular mechanism governing such occurrence. Here we report that in chronically infected mice, the splenic cDC1-cDC2 balance shifted toward the cDC2 subtype and that the receptor T cell immunoglobulin and mucin protein-3 (TIM-3) expressed by DCs played a key role in mediating this effect. Transfer of TIM-3-silenced DCs in fact prevented the predominance of the cDC2 subtype in mice with chronic LD infection. We also found that LD actually upregulated TIM-3 expression on DCs by triggering a TIM-3-mediated signaling pathway STAT3 (signal transducer and activator of transcription 3)→interleukin (IL)-10→c-Src→transcription factors Ets1, Ets2, USF1, and USF2. Notably, TIM-3 promoted STAT3 activation via a non-receptor tyrosine kinase Btk. Adoptive transfer experiments further demonstrated a critical role for STAT3-driven TIM-3 upregulation on DCs in increasing cDC2 abundance in chronically infected mice, which ultimately aided disease pathogenesis by augmenting Th2 responses. These findings document a new immunoregulatory mechanism contributing to disease pathology during LD infection and define TIM-3 as a key mediator of this process.

## Introduction

Visceral leishmaniasis (VL), caused by *Leishmania donovani* (LD), is the second most fatal parasitic disease in the world after malaria [1]. The protective immune activity is largely controlled by dendritic cells (DCs), the prime initiators of antileishmanial T cell responses [2–4]. In fact, during LD infection, DCs serve as an early source of interleukin (IL)–12 [5]. The latter skews the differentiation of naïve T cells to host-protective type-1 T helper (Th1) cells [2]. Strikingly, the immunosuppressive cytokine IL-10, which is expressed during active VL and contributes to disease pathogenesis, is also produced by DCs [6, 7]. Therefore, DC production of IL-12 versus IL-10 serves as a critical factor in determining the disease outcome. Normally, IL-12 is produced in large amounts by type-1 conventional DCs (cDC1) [8]. In contrast, IL-12 is produced in lower amounts by type-2 conventional DCs (cDC2) [9]. Interestingly, cDC2 also produces IL-10 upon stimulation [10, 11]. Moreover, cDC1 and cDC2 preferentially drive Th1 and Th2 responses, respectively [9, 12]. Based on these reports, we speculated that LD possibly influences the cDC1-cDC2 balance, which then perhaps determines the disease outcome. However, it is currently unknown whether *Leishmania* infection affects the cDC1-cDC2 balance and the molecular mechanism by which *Leishmania* mediates such an effect.

In the recent past, T cell immunoglobulin and mucin protein-3 (TIM-3) has emerged as important immunoregulatory receptor [13]. TIM-3 was originally identified as a Th1-specific surface protein [14]. Later on, TIM-3 was also found to be expressed by DCs [15]. A recent study further confirmed that TIM-3 is expressed on both cDC1 and cDC2 [8]. Previously, we have shown that TIM-3 impairs IL-12 production by DCs [16]. Furthermore, some investigators have documented the role of TIM-3 in inducing IL-10 production, albeit in macrophages [17]. These reports led us to speculate that TIM-3, although expressed by both cDC1 and cDC2, may favor in vivo dominance of the cDC2 subtype.

Recently, a few reports have depicted an enhanced TIM-3 expression on CD8^+^ T cells in visceral and cutaneous leishmaniasis patients [18, 19]. However, it is still unknown whether and how LD regulates TIM-3 expression on DCs. Furthermore, the role of TIM-3 in regulating the cDC1-cDC2 balance remains unclear. In fact, the immunoregulatory role of TIM-3 during leishmaniasis is not yet well understood. Herein we, therefore, asked: whether the cDC1-cDC2 balance is perturbed during chronic LD infection; and, if so, whether TIM-3 plays any role in mediating this process; whether and how LD regulates TIM-3 expression on DCs; and finally assessed the significance of the molecular pathway, via which LD controls TIM-3 expression on DCs, in increasing the abundance of any specific DC subtype in vivo.

## Results

### LD infection increases cDC2 number in mice in a TIM-3-dependent manner

**Fig. 1:**
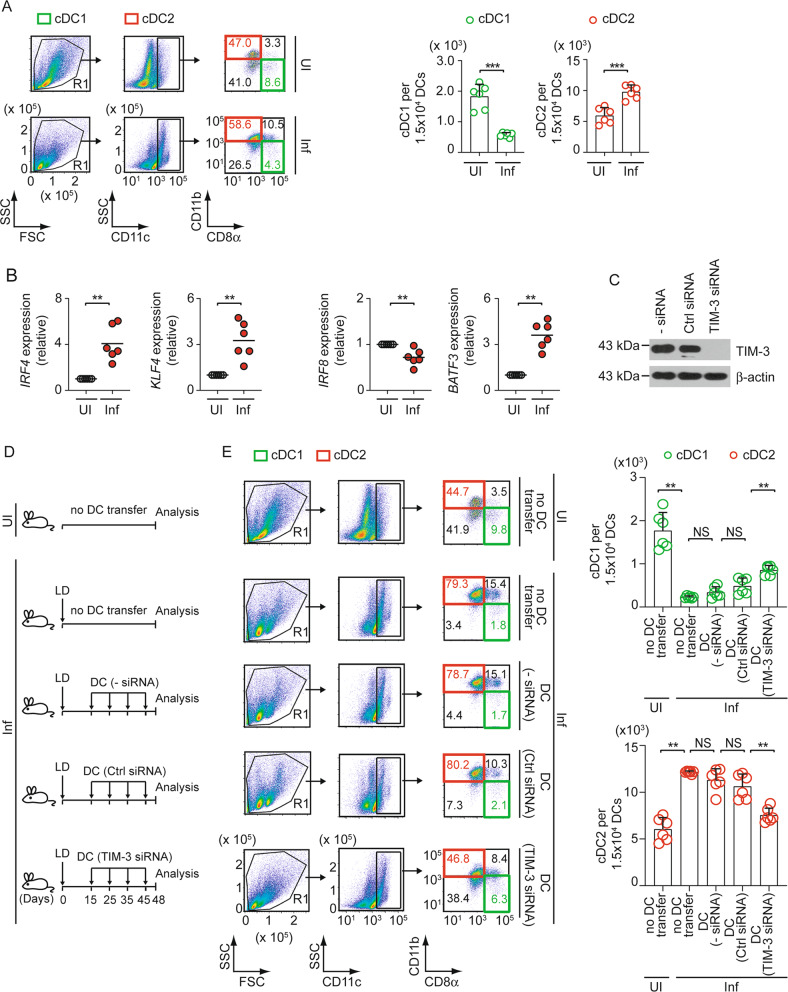
TIM-3 expressed by DCs promotes cDC2 abundance in chronically infected mice. **A** The frequency (left) and number [per 1.5 × 10^4^ DCs (i.e., CD11c^+^ gated cells); right] of cDC1 (CD11c^+^CD11b^−^CD8α^+^) and cDC2 (CD11c^+^CD11b^+^CD8α^−^) in splenocytes prepared from uninfected (UI) and LD-infected (Inf) mice; assessed on day 45 postinfection by flow cytometry (representative data of *n* = 6; left). Numbers in quadrants indicate the percent cells in each throughout. Right, bar graphs show compiled data (*n* = 6 mice per group). **B** Expression of *IRF4*, *KLF4*, *IRF8*, and *BATF3* mRNA in sDCs (i.e., CD11c^+^ spleen cells) that were magnetically sorted from splenocytes of uninfected and 45-day-infected mice; analyzed by quantitative RT-PCR. Results were normalized to the expression of *ACTB* mRNA (encoding β-actin) and are presented as fold change relative to sDCs of uninfected mice (*n* = 6 replicates per group). **C** A representative immunoblot (out of *n* = 2) showing the expression of TIM-3 and β-actin (loading control) in BMDCs left untransfected (−siRNA) or transfected with control (Ctrl) siRNA or TIM-3-specific siRNA. **D** Experimental schematic for adoptive transfer of TIM-3-silenced BMDCs: BALB/c BMDCs (1 × 10^6^) were transfected with siRNAs as in panel (**C**) and then transferred intravenously into LD-infected BALB/c mice on indicated days postinfection. Alternatively, both uninfected and LD-infected mice were left without any DC transfer. On day 48 postinfection, the spleens of these mice were isolated for analyses indicated in panel (**E**). **E** The frequency (left) and number (per 1.5 × 10^4^ CD11c^+^ DCs; right) of cDC1 and cDC2 in splenocytes of uninfected and LD-infected mice treated as in panel (**D**); measured via flow cytometry on day 48 postinfection. Left panel, representative data of *n* = 6. Right panel, compiled data (*n* = 6 mice per group). See Supplementary Fig. 2A, which shows the efficiency of TIM-3 silencing in BMDCs localized in the spleens of recipient mice after one day of transfer. Each symbol in (**A**) (right panel), (**B**), and (**E**) (right panel) represents data of an individual mouse; bars (in panel (**B**)) indicate means. Error bars indicate SD. ^**^p < 0.01, ^***^p < 0.001; NS, not significant.

Because the ability of LD to influence the cDC1-cDC2 balance remains unaddressed, we first investigated this aspect. Accordingly, we compared the abundance of cDC1 (CD11c^+^CD11b^−^CD8α^+^) and cDC2 (CD11c^+^CD11b^+^CD8α^−^) [20, 21] in the spleens of uninfected versus LD-infected mice by measuring the frequency and number of these DC subtypes via flow cytometry. In this regard, we used 45-day-infected mice because we wanted to determine the predominance of the cDC1 or cDC2 subtype during the chronic stage of LD infection (>28 days; [2, 22]). Furthermore, a significant Th2 response correlated with increased parasite load has been reported in the spleens of LD-infected mice during 40 to 48 days postinfection [23, 24]. Notably, the Th2 response is known to be promoted by cDC2 [9, 12, 20]. Therefore, it was quite intriguing to investigate whether LD favors the cDC2 abundance during chronic infection. In comparison to uninfected mice, LD-infected mice demonstrated a considerable reduction in cDC1 cells and a significant increase in cDC2 cells (Fig. 1A). We then performed quantitative RT-PCR to compare the mRNA expression of cDC1-specific transcription factors IRF8 and BATF3 versus cDC2-specific transcription factors IRF4 and KLF4 [25] in splenic DCs (sDCs; defined by CD11c^+^ cells [26–31]) magnetically sorted from uninfected or 45-day-infected mice. As compared to uninfected mice, sDCs from LD-infected mice exhibited higher expression of *IRF4* and *KLF4* and lower expression of *IRF8* (Fig. 1B). Surprisingly, we found more *BATF3* (another cDC1-specific transcription factor) expression in sDCs from LD-infected mice than those from uninfected mice (Fig. 1B). However, this elevated *BATF3* expression in LD-infected mice failed to raise the abundance of cDC1 due to reduced *IRF8* expression [32, 33]. Accordingly, LD preferentially increased the cDC2 population in vivo.

Studies have shown that cDC2 promotes immune tolerance partly via an inhibitory receptor DCIR2 [34, 35]. The latter is critically required for the maintenance of cDC2 as well [36]. Likewise, TIM-3 also serves as an inhibitory receptor on DCs [16]. However, whether DC-expressed TIM-3 has any role in regulating cDC1-cDC2 balance is currently unknown. To explore this possibility, we suppressed (or not) TIM-3 expression in bone marrow-derived DCs (BMDCs) by siRNA (Fig. 1C). We then adoptively transferred these cells into LD-infected syngeneic mice on indicated days postinfection (Fig. 1D). On the 48th day after LD infection, we finally determined the frequency and number of splenic cDC1 and cDC2 in above-treated mice. Relative to uninfected mice, LD-infected mice showed a reduced cDC1 population and an increased cDC2 population in the spleen (Fig. 1E). In contrast, adoptive transfer of TIM-3-silenced BMDCs increased cDC1 abundance and decreased cDC2 abundance in the spleens of LD-infected mice (Fig. 1E). Notably, we did not specifically examine here whether exogenously transferred TIM-3-silenced BMDCs or endogenous sDCs underwent cDC1 conversion. Instead, we wanted to see if transferring TIM-3-silenced BMDCs affected the overall frequency of cDC1 and cDC2 in the spleens of chronically infected mice. In this regard, we observed that the proportion of BMDCs localizing to the spleens of recipient mice after each adoptive transfer was very small compared to the total number of sDCs (e.g., on days 1, 3, and 7 following BMDC transfer; these proportions were 3.15%, 1.27%, and 0.68% of total sDCs, respectively; Supplementary Fig. 1). Moreover, the adoptively transferred BMDCs stayed in the spleen for only a short time (up to 3 days; Supplementary Fig. 1). These findings set the stage to figure out whether or not the increased cDC1 population in LD-infected mice spleen was derived from exogenously transferred TIM-3-silenced BMDCs. A comparison of our results in Fig. 1E and Supplementary Fig. 1 revealed that the frequency of transferred BMDCs in spleens was lower than the changes in splenic cDC1/cDC2 frequency observed after TIM-3-silenced BMDC transfer in LD-infected mice. For example, after three days of the adoptive transfer, the proportion of BMDCs located in the spleen was only 1.27% of total sDCs (Supplementary Fig. 1). In contrast, at the same time point after TIM-3-silenced BMDC transfer, the frequency of cDC1 increased by 4.2% (compared to control siRNA-transfected BMDC transfer) in LD-infected mice (Fig. 1E). Of note, the three-day interval mentioned here refers to the time gap between the last BMDC transfer (45 days postinfection) and the final analysis of cDC1/cDC2 frequency in LD-infected mice (48 days postinfection; Fig. 1D). Based on these results, the increased splenic abundance of cDC1 and decreased splenic abundance of cDC2 in LD-infected mice after adoptive transfer of TIM-3-silenced BMDCs is most likely due to polarization of endogenous sDCs to cDC1 subtype. We further ascertained the TIM-3 silencing efficiency in BMDCs after adoptive transfer. For this, we transfected BMDCs with control siRNA or TIM-3-specific siRNA, labeled them with eFluor 670 dye, and injected these BMDCs into syngeneic mice. We then evaluated the efficiency of TIM-3 knockdown in eFluor 670^+^ BMDCs (i.e., transferred DCs) that settled in the spleens of recipient mice one day after BMDC transfer by flow cytometry. We found that TIM-3 expression was suppressed in approximately 60% of spleen-localized eFluor 670^+^ BMDCs (Supplementary Fig. 2A).

Now, a question may arise whether the low frequency of spleen-localized transferred BMDCs can influence the splenic cDC1-cDC2 balance in LD-infected mice. Considering this, we adoptively transferred TIM-3-silenced BMDCs multiple times in the above experiments (Fig. 1D, E) to achieve a sustained and significant impact of these transferred DCs on the cDC1-cDC2 balance in LD-infected recipient mice. Our results (Fig. 1D, E) demonstrated that repeated transfer of TIM-3-silenced BMDCs reduces the cDC2 population in the spleens of chronically infected mice. Thus, TIM-3 on DCs plays a pivotal role in promoting cDC2 abundance during chronic LD infection.

### LD upregulates TIM-3 expression on DCs

**Fig. 2:**
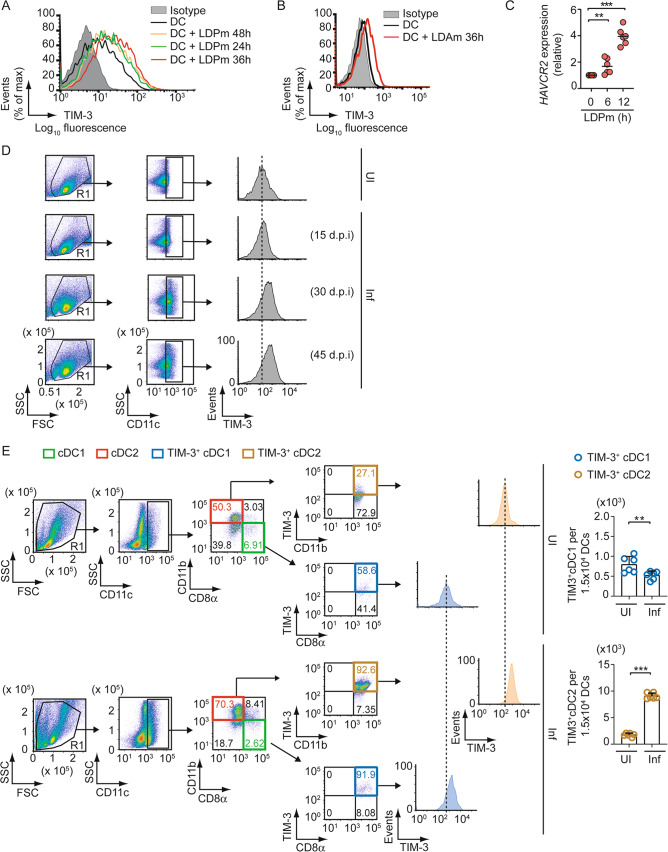
LD upregulates TIM-3 expression on DCs. **A** Flow cytometry analysis (representative data of *n* = 3) of TIM-3 expression on BMDCs following LDPm infection for indicated times. Isotype, isotype control antibody. Compiled data of three replicates for relative mean fluorescence intensities (MFIs) of TIM-3 expression are shown in Supplementary Fig. 3A. **B** Effect of LDAm infection (for 36 h) on surface expression of TIM-3 on BMDCs; analyzed by flow cytometry (representative data of *n* = 3). See Supplementary Fig. 3B for relative MFI data of TIM-3 expression compiled from three replicates. **C** Expression of *HAVCR2* mRNA in BMDCs infected with LDPm for various times. Results were normalized to the expression of *ACTB* mRNA (encoding β-actin) and are presented as fold change relative to uninfected BMDCs (0 h). Combined data of *n* = 6 replicates are shown here. **D** Expression of TIM-3 on sDCs (i.e., CD11c-gated spleen cells) from uninfected mice or LD-infected mice, analyzed via flow cytometry on indicated days postinfection (d.p.i). Data are representative of *n* = 6. The relative MFI of TIM-3 expression (compiled data of *n* = 6 mice per group) is shown in Supplementary Fig. 3C. **E** The level of TIM-3 expression on cDC1 and cDC2 [histograms (left panel); a representative data out of *n* = 6], and the number of TIM-3^+^ cDC1 and TIM-3^+^ cDC2 per 1.5 × 10^4^ CD11c^+^ DCs [right panel; a combined data of *n* = 6 mice per group] in splenocytes of uninfected and LD-infected mice were determined by flow cytometry on day 45 postinfection. The relative MFI of TIM-3 expression on cDC1 and cDC2 (pooled data from *n* = 6 mice per group) has been given in Supplementary Fig. 3D. The gating strategy for TIM-3 has been illustrated in Supplementary Fig. 3E. Each symbol in (**C**) and (**E**) (right panel) denotes data of an individual replicate or mouse, respectively; bars indicate means. Error bars indicate SD. ^**^p < 0.01, ^***^p < 0.001.

Having found that TIM-3 is critical for increased cDC2 abundance during chronic LD infection, we asked whether LD influences TIM-3 expression on DCs. For this purpose, we infected BMDCs with LD promastigotes (LDPm; the extracellular form of LD) for various times and analyzed TIM-3 expression via flow cytometry. The expression of TIM-3 started increasing within 24 h after LDPm infection, peaked at 36 h, and then reduced at 48 h (Fig. 2A; Supplementary Fig. 3A). We detected TIM-3 upregulation on BMDCs also after infection with LD amastigotes (LDAm; the intracellular form of LD) (Fig. 2B; Supplementary Fig. 3B). Consistent with these findings, BMDC infection with LDPm considerably increased *HAVCR2* mRNA expression (*HAVCR2* encodes TIM-3; Fig. 2C). We then determined whether LD influences TIM-3 expression on sDCs in vivo. Temporal analysis showed that TIM-3 expression was upregulated on sDCs as early as 30 days postinfection and sustained up to at least 45 days (Fig. 2D; Supplementary Fig. 3C). These results suggest that LD upregulates TIM-3 on DCs.

We further compared TIM-3 expression on splenic cDC1 and cDC2 from uninfected versus 45-day-infected mice. Relative to uninfected mice, LD-infected mice showed increased TIM-3 expression on both cDC1 and cDC2 (Fig. 2E, histogram panel; Supplementary Fig. 3D, E). Furthermore, in LD-infected mice, TIM-3 expression was higher on cDC1 than on cDC2 (Fig. 2E, histogram panel; Supplementary Fig. 3D, E). However, the splenic abundance of TIM-3-expressing cDC1 was lower, and that of TIM-3-expressing cDC2 was substantially higher in LD-infected mice when compared to uninfected mice (Fig. 2E, FACS dot plots and graphs; Supplementary Fig. 3E). Thus, although LD promotes TIM-3 upregulation more on cDC1, it simultaneously reduces TIM-3-expressing cDC1 population in infected mice. In contrast, LD moderately increases TIM-3 expression on cDC2. But the splenic abundance of the TIM-3-expressing cDC2 subtype increases substantially after LD infection.

### LD-stimulated TIM-3 upregulation requires STAT3-driven IL-10 production mediated by the TIM-3-Btk axis

**Fig. 3:**
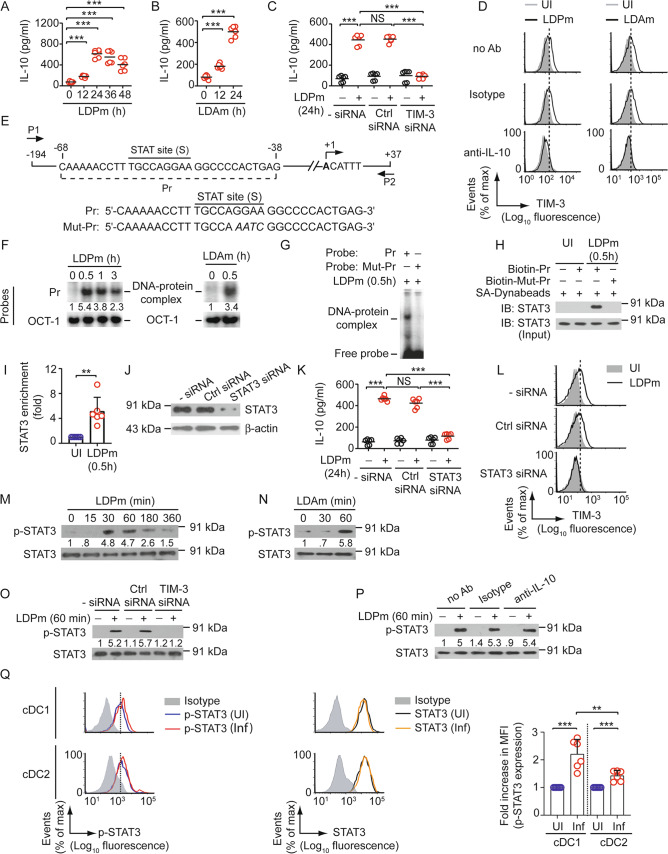
TIM-3-dependent LD-induced IL-10 production via STAT3 promotes TIM-3 upregulation on DCs. **A**–**C** BMDCs were infected with LDPm (**A**, **C**) or LDAm (**B**) for specified times. In some experiments (**C**), BMDCs were transfected with indicated siRNAs before LDPm infection. IL-10 production by BMDCs was measured by ELISA (compiled data of *n* = 6 replicates). −siRNA and Ctrl siRNA represent BMDCs left untransfected or transfected with control siRNA, respectively. **D** Flow cytometry analysis of TIM-3 expression on BMDCs left uninfected (UI) or infected for 36 h with LDPm (left panel) or LDAm (right panel) in the absence [no antibody (Ab)] or presence of isotype control antibody (isotype) or anti-IL-10 antibody. **E** Schematic of the mouse *IL-10* promoter depicting the location of putative STAT site (S) and ChIP primers (P1 and P2), and details of oligonucleotides used for EMSA and DNA pull-down assay. Mouse *IL-10* promoter-specific Pr and Mut-Pr oligonucleotides contain wild-type and mutated (mutations are indicated by italics) STAT sites, respectively. **F**, **G** EMSA of nuclear extracts of BMDCs infected with LDPm (**F**, left panel; and **G**) or LDAm (**F**, right panel) for specified times; assayed with indicated probes. Numbers below lanes in panel (**F**), densitometry [normalized to OCT-1 binding (internal control)]; presented relative to that of uninfected BMDCs (0 h). **H** Nuclear proteins of uninfected or LDPm-infected BMDCs (infection was done for 0.5 h) that interacted with specified biotin-labeled oligonucleotides were pulled down using streptavidin (SA)-coupled Dynabeads and then immunoblotted (IB) for indicated proteins. Input, nuclear extracts (5%) before pull-down. **I** Recruitment of STAT3 to the mouse *IL-10* promoter in BMDCs infected with LDPm (for 0.5 h) or kept uninfected was assessed by ChIP-quantitative PCR analysis. Results are presented as fold enrichment relative to uninfected BMDCs (compiled data of *n* = 6 replicates). **J** BMDCs were transfected or not (−siRNA) with control siRNA or STAT3 siRNA and then immunoblotted for STAT3 and β-actin (loading control). **K** IL-10 production by BMDCs that had been transfected with siRNAs as in panel (**J**) and infected for 24 h with LDPm (+) or kept uninfected (−); measured by ELISA (compiled data of *n* = 6 replicates). **L** Analyzing (by flow cytometry) the effect of STAT3 silencing on TIM-3 expression by BMDCs infected with LDPm for 36 h or left uninfected. **M**, **N** BMDCs were infected with LDPm (**M**) or LDAm (**N**) for various times and immunoblotted for total or phosphorylated (p-) STAT3. Below lane, densitometry (normalized to total STAT3); presented relative to uninfected BMDCs (0 h). **O** Expression of total and phosphorylated STAT3 in BMDCs transfected with specified siRNAs and then infected (+) or not (−) with LDPm for 60 min, examined by immunoblot analysis. Below lane, relative densitometry as in panel (**M**). **P** Immunoblot analysis of total and phosphorylated STAT3 in BMDCs kept uninfected (−) or infected (+) with LDPm for 60 min in the absence (no Ab) or presence of specified antibodies (above lanes). Below lane, relative densitometry as in panel (**M**). **Q** Intracellular expression of phosphorylated STAT3 and total STAT3 in cDC1 and cDC2 of uninfected and LD-infected mice; analyzed by flow cytometry on the 45th day postinfection (left panel; representative data out of *n* = 6). Right panel, corresponding MFIs of phosphorylated STAT3 expression have been shown as bar graphs (pooled data from *n* = 6 mice per group). The MFIs of phosphorylated STAT3 and total STAT3 were calculated after subtracting the background signal (isotype control). Then, the MFIs of phosphorylated STAT3 were normalized to those of total STAT3 values and are presented as fold change relative to uninfected DCs. Data are representative of *n* = 3 (**D**, **F**, **H**, **L**, **M**, **O**, and **P**) or *n* = 2 (**G**, **J**, and **N**). Each symbol in panels (**A**–**C**), (**I**), and (**K**) denotes data of an individual replicate, and that in panel (**Q**) corresponds to data of an individual mouse. The horizontal bars in (**A**–**C**) and (**K**) indicate means. Compiled data (*n* = 3) of densitometry analyses for (**F**), (**M**), (**O**), and (**P**) panels and relative MFIs of TIM-3 expression for (**D**) and (**L**) panels are presented in Supplementary Fig. 4. Error bars indicate SD. ^**^p < 0.01, ^***^p < 0.001; NS, not significant.

Next, we determined the mechanism by which LD upregulates TIM-3 expression on DCs. Earlier, we have shown that TIM-3 is upregulated on DCs upon IL-10 treatment [37]. Notably, IL-10 is reported to be expressed at an elevated level in advanced VL patients [6]. Therefore, we speculated a role for IL-10 in LD-induced TIM-3 upregulation on DCs. Initially, we tested whether LD induces IL-10 secretion from DCs. Kinetic analyses showed that IL-10 production was augmented within 12 h after LDPm infection and reached a maximum at 24–36 h (Fig. 3A). Similarly, LDAm stimulated IL-10 secretion from BMDCs (Fig. 3B). The LD-induced IL-10 production, however, was blocked by TIM-3 silencing in BMDCs (Fig. 3C). Then, we determined the functional role for IL-10 in LD-mediated TIM-3 upregulation on DCs. We observed that neutralization of IL-10 with anti-IL-10 antibody prevented TIM-3 upregulation induced by LDPm or LDAm (Fig. 3D; Supplementary Fig. 4A). These data suggest that LD triggers IL-10 production by DCs via TIM-3 and that IL-10 subsequently promotes TIM-3 upregulation on DCs.

We afterward determined how LD augmented IL-10 secretion from DCs. For this, we analyzed the mouse *IL-10* promoter sequence with the TFBIND program and identified a putative binding site for the STAT transcription factors (S site: ^-58^TGCCAGGAA^-50^; base positions are relative to transcription initiation site) (Fig. 3E). In fact, the ^-58^TGCCAGGAA^-50^ sequence is reported to bind STAT3 [38]. Thus, it seemed likely that the ^-58^TGCCAGGAA^-50^ sequence serves as a STAT3-binding site in the mouse *IL-10* promoter. Notably, the STAT3-binding sequence in mouse *IL-10* promoter remains unidentified, although STAT3 is known to regulate *IL-10* expression [39]. Accordingly, we investigated whether LD induced STAT3 binding to the mouse *IL-10* promoter in DCs. Electrophoretic mobility shift assay (EMSA) demonstrated that BMDC infection with LDPm or LDAm induced the binding of nuclear protein (or proteins) to mouse *IL-10* promoter-specific Pr probe, which contained the S site (Fig. 3E, F; Supplementary Fig. 4B). Mutation at the S site, however, blocked this nuclear protein binding (Fig. 3G). DNA pull-down assay showed that the biotinylated Pr oligonucleotide successfully precipitated STAT3 from nuclear preparations of LDPm-infected BMDCs, but the same oligonucleotide containing mutated S site (Mut-Pr) did not (Fig. 3H). Chromatin immunoprecipitation (ChIP) assays further demonstrated enhanced recruitment of STAT3 to the *IL-10* promoter upon LDPm infection (Fig. 3I). Thus, LD induces STAT3 binding to the *IL-10* promoter in DCs.

Next, we performed a reporter assay to examine whether STAT3 transactivates the mouse *IL-10* promoter. Overexpression of STAT3 in HEK293T cells strongly enhanced the wild-type *IL-10* promoter activity. This STAT3-driven *IL-10* promoter activity was drastically reduced when the STAT3 site in the *IL-10* promoter was mutated (Supplementary Fig. 5). Moreover, STAT3 silencing by siRNA impaired LD-stimulated IL-10 secretion and subsequent TIM-3 upregulation on BMDCs (Fig. 3J–L; Supplementary Fig. 4C). We also noted that infection with LDPm or LDAm induced STAT3 phosphorylation in BMDCs (Fig. 3M, N; Supplementary Fig. 4D). Such effect, however, was blocked by TIM-3 silencing (Fig. 3O; Supplementary Fig. 4E). In contrast, antibody-mediated neutralization of IL-10 did not affect LD-induced STAT3 phosphorylation (Fig. 3P; Supplementary Fig. 4F). Likewise, transfection of IL-10-specific siRNA successfully reduced LD-stimulated IL-10 expression in DCs without impacting LD-induced STAT3 phosphorylation (Supplementary Fig. 6). Thus, LD promotes STAT3 activation in DCs in a TIM-3-dependent but IL-10-independent manner. We then compared the level of phospho-STAT3 in cDC1 and cCD2 of uninfected and 45-day-infected mice. Relative to uninfected mice, LD-infected mice showed a pronounced increase in phospho-STAT3 expression in the cDC1 subtype. Comparatively, phospho-STAT3 expression was slightly increased within cDC2 in LD-infected mice (Fig. 3Q). Together, these data suggest that STAT3 mediates LD-induced TIM-3 upregulation on DCs by inducing IL-10 secretion.

After this, we sought to identify the upstream activator of STAT3. One of the potential signaling mediators appeared to be Bruton’s tyrosine kinase (Btk), a Tec non-receptor tyrosine kinase family member. This is because Btk has been reported to induce STAT3 activation and consequent IL-10 production in B cells [upon induction with Epstein-Barr Virus Latent Membrane Protein 2A (LMP2A)] and leukemic CLL cells [40, 41]. Furthermore, our recent study [24] has shown that LD triggers TIM-3 signaling (as measured by TIM-3 phosphorylation) in DCs and that this LD-induced TIM-3 recruits and activates Btk. Btk then mediates LD-induced IL-10 production by DCs [24]. Based on all these reports, we investigated whether Btk was involved in STAT3-mediated TIM-3 upregulation on DCs. To explore this possibility, we analyzed the effect of Btk silencing on LD-induced STAT3 activation, binding of STAT3 to the *IL-10* promoter, and TIM-3 upregulation on DCs. We observed that all these LD-induced events were inhibited by Btk silencing in DCs (Supplementary Fig. 7). These data, together with our previous study [24], suggest that Btk acts as an upstream activator of STAT3, thereby promoting STAT3-mediated IL-10 production and subsequent TIM-3 upregulation on DCs during LD infection. Our previous report has also documented that the blockade of TIM-3 with anti-TIM-3 antibody (clone RMT3-23) prevents LD-induced TIM-3 phosphorylation in DCs [24]. Here we found that pretreatment with the same anti-TIM-3 antibody prevented LD-induced Btk and STAT3 phosphorylation and upregulation of TIM-3 expression on DCs (Supplementary Fig. 8A–C). Notably, this TIM-3 blocking antibody did not interfere with subsequent TIM-3 immunostaining with fluorochrome-conjugated anti-TIM-3 antibody (clone B8.2C12; Supplementary Fig. 8D). Thus, the inhibition of LD-induced TIM-3 upregulation observed after anti-TIM-3 antibody treatment was not due to reduced immunostaining of TIM-3 caused by the antibody-mediated blockade. Together, these results suggest that LD promotes TIM-3 upregulation on DCs by inducing STAT3-mediated IL-10 production through the TIM-3-Btk axis.

### LD-induced IL-10 upregulates TIM-3 on DCs via the c-Src-Ets, USF pathway

**Fig. 4:**
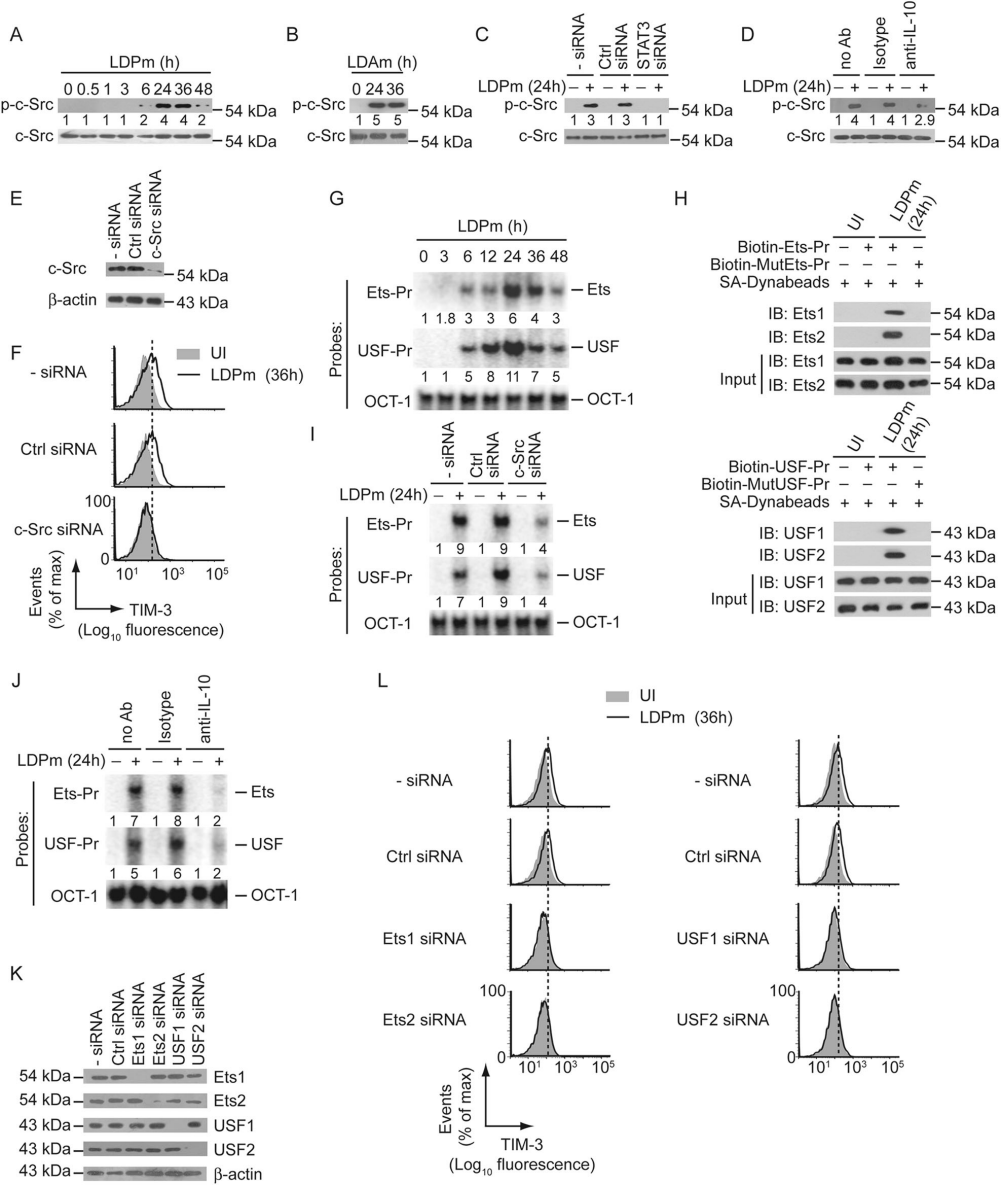
LD-induced IL-10 production upregulates TIM-3 on DCs via the c-Src-Ets, USF pathway. **A**, **B** Immunoblot analysis of total and phosphorylated c-Src expression in BMDCs infected with LDPm (**A**) or LDAm (**B**) for indicated times. Numbers below lanes, densitometry; normalized to total c-Src and presented relative to BMDCs left uninfected (0 h). **C**, **D** Analyzing (via immunoblot analysis) the effect of STAT3 silencing (**C**; by STAT3-specific siRNA) and neutralization of IL-10 (**D**; with anti-IL-10 antibody) on the expression of phosphorylated c-Src in BMDCs infected with LDPm for 24 h (+) or left uninfected (−). Expression of total c-Src serves as a loading control. Numbers below lanes, densitometry as in panel (**A**); presented relative to control BMDCs [BMDCs left untransfected (−siRNA) and uninfected (**C**); or BMDCs left uninfected and cultured without any antibody (no Ab) (**D**)]. **E** Immunoblot analysis of c-Src and β-actin (loading control) expression in BMDCs kept untransfected (−siRNA) or transfected with control siRNA or c-Src-specific siRNA. **F** BMDCs were transfected with siRNAs as in panel (**E**) and then infected with LDPm for 36 h or left uninfected. The expression of TIM-3 on BMDCs was evaluated via flow cytometry. **G** BMDCs were infected with LDPm for indicated times. EMSA was performed using nuclear extracts of these BMDCs and the mouse *HAVCR2* promoter-specific Ets-Pr and USF-Pr probes that contained Ets- and USF-binding sites, respectively (Ets-Pr and USF-Pr were previously described as Pr1 and Pr2 probes, respectively, in ref. [37]). OCT-1 binding serves as an internal control. Numbers below lanes, densitometry; normalized to OCT-1 binding and presented relative to uninfected BMDCs (0 h). **H** Nuclear proteins of uninfected BMDCs or 24 h LDPm-infected BMDCs were subjected to pull-down assay using indicated biotin-labeled oligonucleotides and streptavidin-conjugated Dynabeads and then immunoblotted for Ets1 and Ets2 (upper panel) or USF1 and USF2 (lower panel). Input, nuclear extracts (5%) before pull-down. **I** Binding of Ets and USF transcription factors to the mouse *HAVCR2* promoter was evaluated via EMSA using probes specified in the left margin and the nuclear extracts from BMDCs that were transfected with indicated siRNAs, and then infected (+) or not (−) with LDPm for 24 h. Numbers below lanes, densitometry as in panel (**G**). **J** EMSA of nuclear extracts of BMDCs that had been kept uninfected (−) or infected with LDPm for 24 h (+) in the presence or absence of indicated antibodies; assayed with indicated probes. Numbers below lanes, densitometry; presented relative to uninfected BMDCs cultured without antibody (no Ab). **K** Immunoblot analysis ascertaining the silencing of Ets1, Ets2, USF1, and USF2 by respective siRNAs in BMDCs. β-actin was used as a loading control. **L** Flow cytometry analysis of TIM-3 expression on BMDCs transfected with various siRNAs (left margin) and then infected with LDPm for 36 h or left uninfected. Data in panels (**A**), (**C**), (**D**), (**F**), (**G**), (**I**), (**J**), and (**L**) are representative of *n* = 3, and data in panels (**B**), (**E**), (**H**), and (**K**) are representative of *n* = 2. Compiled data (*n* = 3) of densitometry results for panels (**A**), (**C**), (**D**), (**G**), (**I**), and (**J**), and relative MFI values of TIM-3 expression for panels (**F**) and (**L**) are shown in Supplementary Fig. 9.

We have previously shown that IL-10 upregulates TIM-3 expression on DCs via the pathway c-Src→transcription factors Ets1, Ets2, USF1, and USF2 [37]. Therefore, we investigated whether IL-10 produced during LD infection utilizes the same pathway to upregulate TIM-3 expression on DCs. Initially, we checked whether LD induces c-Src activation (assessed by c-Src Tyr416 phosphorylation [37]) in DCs; and, if so, whether this c-Src activation depends on STAT3-driven IL-10 production by DCs. Immunoblot analysis showed that c-Src phosphorylation was triggered at 24–36 h after LDPm infection (Fig. 4A, Supplementary Fig. 9A). Likewise, LDAm induced c-Src phosphorylation in BMDCs (Fig. 4B). Silencing of STAT3 in BMDCs or neutralization of IL-10 with anti-IL-10 antibody, however, blocked this effect (Fig. 4C, D; Supplementary Fig. 9B, C). Thus, LD-induced STAT3-mediated IL-10 production promotes c-Src activation in DCs. Importantly, c-Src silencing by siRNA prevented LDPm-induced TIM-3 upregulation on DCs (Fig. 4E, F; Supplementary Fig. 9D). This finding suggests an essential role for c-Src in LD-stimulated TIM-3 upregulation on DCs.

We next determined whether c-Src or its inducer IL-10 promoted LD-induced TIM-3 upregulation on DCs via Ets and USF. EMSA and DNA pull-down assay showed that BMDC infection with LDPm induced Ets1, Ets2, USF1, and USF2 binding to the mouse *HAVCR2* promoter (Fig. 4G, H; Supplementary Fig. 9E). However, silencing of c-Src or neutralization of IL-10 (with anti-IL-10 antibody) blocked this effect (Fig. 4I, J; Supplementary Fig. 9F, G). Furthermore, silencing of Ets1, Ets2, USF1, or USF2 attenuated LDPm-stimulated TIM-3 upregulation on BMDCs (Fig. 4K, L; Supplementary Fig. 9H). Therefore, LD-induced STAT3-mediated IL-10 production promotes TIM-3 upregulation on DCs via the c-Src-Ets, USF pathway.

### STAT3-TIM-3 axis promotes splenic cDC2 abundance during chronic LD infection

**Fig. 5:**
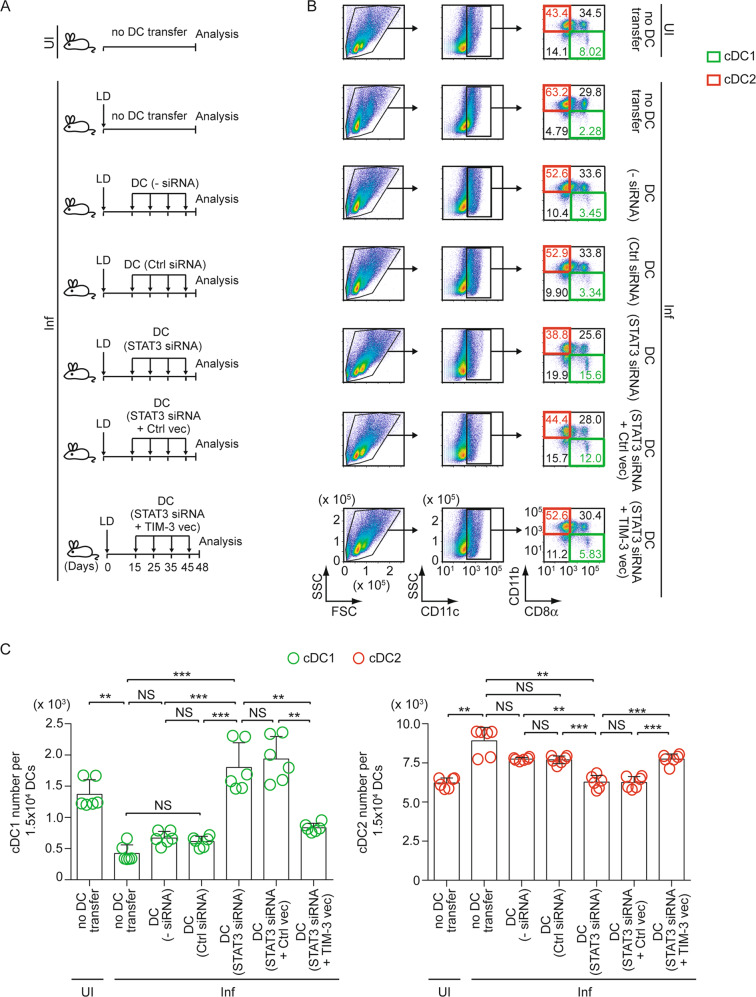
The STAT3-TIM-3 axis promotes cDC2 abundance in chronically LD-infected mice. **A** Experimental procedure for DC adoptive transfer: BALB/c BMDCs (1 × 10^6^) were transfected (or not) with control siRNA or with STAT3-specific siRNA either alone or together with control vector (Ctrl vec) or TIM-3-expressing vector (TIM-3 vec) (see Supplementary Fig. 10 demonstrating the level of TIM-3 on these cells). BMDCs (1 × 10^6^) were then transferred intravenously into LD-infected BALB/c mice on specified days postinfection (indicated by arrows). On the 48th day postinfection, the spleen and liver of these LD-infected mice were isolated for various analyses [(**B**) and (**C**) panels of this figure, and Figs. 6 and 7]. The efficiency of STAT3 silencing in BMDCs localized to the spleens of recipient mice one day after the transfer is shown in Supplementary Fig. 2B. **B**, **C** The frequency (**B**) and the number (per 1.5 × 10^4^ CD11c^+^ sDCs; **C**) of cDC1 and cDC2 in the spleens of mice treated as in panel (**A**), analyzed by flow cytometry on the 48th day postinfection. A representative (*n* = 6) flow cytometry data is presented in panel (**B**), and compiled data of *n* = 6 mice per group are presented in panel (**C**). Error bars indicate SD. ^**^p < 0.01, ^***^p < 0.001; NS, not significant.

**Fig. 6:**
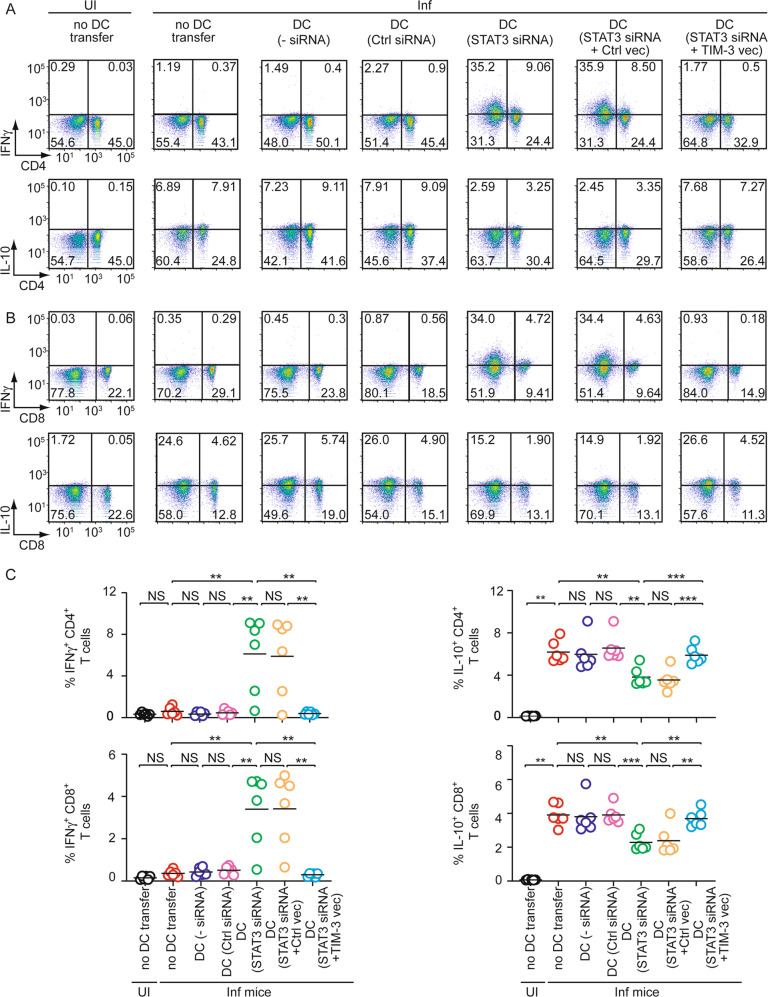
The STAT3-TIM-3 axis augments the Th2 response in LD-infected mice. Control-silenced BMDCs or STAT3-silenced BMDCs (with or without TIM-3 overexpression) were adoptively transferred into LD-infected mice (see Fig. 5 legend for experimental details). On the 48th day postinfection, the splenocytes were prepared from these mice for various analyses indicated in panels (**A**–**C**). **A**, **B** The frequency of CD4^+^ (**A**) and CD8^+^ (**B**) T cells expressing IFNγ or IL-10 was evaluated by flow cytometry (representative data of *n* = 6). **C** Cumulative data of *n* = 6 mice per group are plotted in the graphs. Each symbol represents the data of an individual mouse, and the bars indicate means. ^**^p < 0.01, ^***^p < 0.001; NS, not significant.

**Fig. 7:**
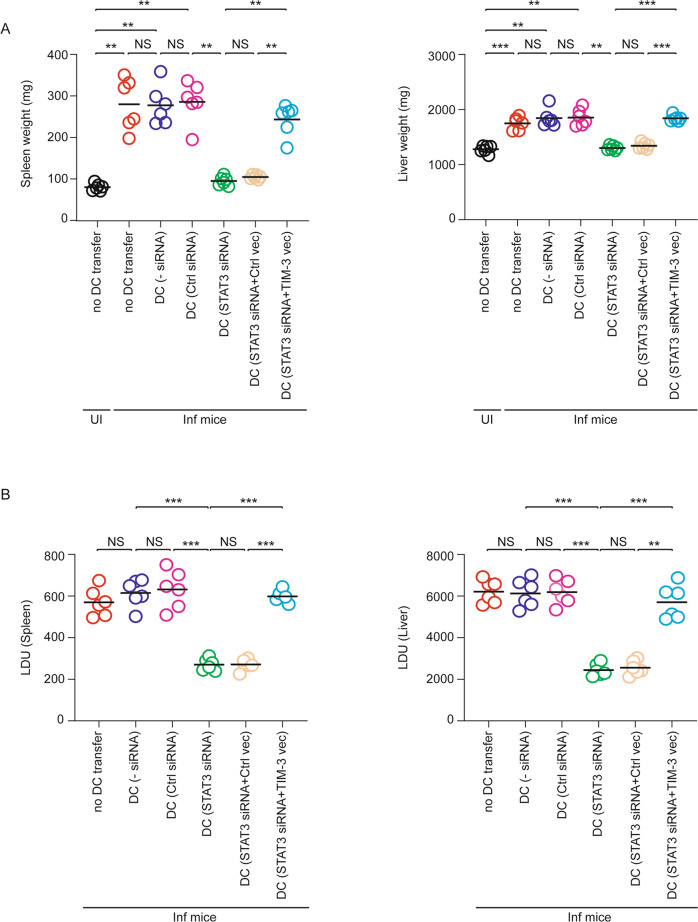
The STAT3-TIM-3 axis mediates disease pathology during LD infection. Adoptive transfer of control-silenced and STAT3-silenced BMDCs (with or without TIM-3 overexpression) into LD-infected mice was performed as described in Fig. 5. The weight of spleens and livers (**A**) and the parasite burden in these organs [expressed as Leishman-Donovan Unit (LDU); **B**] of above-described treated mice were assessed on the 48th day postinfection and are plotted graphically. Compiled data of *n* = 6 mice per group are presented here. Each symbol corresponds to the data of an individual mouse, and the bars indicate means. ^**^p < 0.01, ^***^p < 0.001; NS, not significant.

Given the demonstration that LD increases cDC2 abundance in a TIM-3-dependent manner and that TIM-3 is upregulated by LD on DCs due to increased STAT3 activity, we investigated the relevance of STAT3-TIM-3 axis in the alteration of splenic cDC1-cDC2 balance and subsequent antileishmanial immune responses during chronic LD infection. For this, we adoptively transferred control or STAT3 siRNA-transfected BMDCs to LD-infected mice on specified days (Fig. 5A). In this regard, we observed that STAT3 expression was suppressed in about 66% of STAT3 siRNA-transfected BMDCs localized within the spleens of recipient mice after one day of transfer (Supplementary Fig. 2B). The latter finding demonstrated the efficiency of STAT3 knockdown in BMDCs upon in vivo administration. In addition to STAT3 siRNA-transfected BMDCs, we used TIM-3-overexpressing STAT3 siRNA-transfected BMDCs for adoptive transfer in some experimental sets (Fig. 5A; Supplementary Fig. 10). On the 48th day postinfection, we determined the splenic abundance of cDC1 and cDC2, the liver and spleen weights and parasite load, and the frequency of splenic CD4^+^ and CD8^+^ T cells producing IFNγ or IL-10. Compared to uninfected mice, LD-infected mice had less cDC1 and more cDC2 population in the spleens (Fig. 5B, C). However, the splenic abundance of cDC1 was markedly increased, and that of cDC2 was considerably reduced in LD-infected mice following the transfer of STAT3 siRNA-transfected BMDCs but not control siRNA-transfected BMDCs (Fig. 5B, C). It is notable that here also we investigated whether the transfer of STAT3 siRNA-transfected BMDCs influences overall cDC1 or cDC2 abundance in the spleens of LD-infected mice rather than examining whether changes in cDC1 or cDC2 frequency arise from adoptively transferred STAT3 siRNA-transfected BMDCs. However, as discussed before for TIM-3-silenced BMDC transfer, the increase in cDC1 frequency (and decrease in cDC2 frequency) in LD-infected mice after STAT3 siRNA-transfected BMDC transfer may be due to the polarization of endogenous sDCs to the cDC1 subtype. Nonetheless, our findings showed that the transfer of STAT3 siRNA-transfected BMDC increased cDC1 abundance (while decreasing cDC2 abundance) in LD-infected mice. Interestingly, these effects of STAT3 siRNA-transfected BMDCs were greatly compromised when we overexpressed TIM-3 in these cells before adoptive transfer (Fig. 5B, C). Corroborating with our above findings, LD-infected mice continued to display enhanced type-2 T cell response, hepatosplenomegaly, and an increased liver and splenic parasite burden regardless of the transfer of control siRNA-transfected BMDCs (Figs. 6, 7). The adoptive transfer of STAT3 siRNA-transfected BMDCs into LD-infected mice, on the other hand, largely attenuated these effects while enhancing type-1 T cell responses. In marked contrast, the transfer of TIM-3-overexpressing STAT3 siRNA-transfected BMDCs reestablished the type-2-dominated T cell response, hepatosplenomegaly, and an increased liver and splenic parasite load in LD-infected mice (Figs. 6, 7). Therefore, the STAT3-TIM-3 axis plays a key role in promoting cDC2 abundance and subsequent type-2 T cell response, which eventually suppresses antileishmanial immunity.

## Discussion

The Th1-Th2 balance plays a pivotal role in determining the disease outcome during LD infection. While the Th1 response has a curative effect, the Th2 response is associated with disease progression [42–44]. Notably, the immune bias toward Th1 or Th2 phenotype is largely controlled by DCs, in addition to other important determinants such as the local cytokine microenvironment [45–47]. It is generally believed that the cDC1 subtype promotes Th1 response, whereas the cDC2 subtype facilitates Th2 activity [9, 12, 20]. However, whether cDC1 and cDC2 mediate such effects during LD infection remains undefined. Even it is currently unknown whether LD promotes DC polarization to any specific subtype in vivo. Therefore, we investigated whether LD infection alters the cDC1-cDC2 balance in vivo and thus affects the antileishmanial immune response and eventual disease pathogenesis. Additionally, we have identified the molecular mechanism and the key receptor mediating such immunoregulatory effects of LD. Three major observations were obtained in this study.

First, our findings show that during chronic LD infection, the splenic cDC1-cDC2 balance shifts toward the cDC2 subtype. In this context, it is noteworthy that *BATF3*-expressing cDC1 confers resistance to VL caused by *Leishmania infantum* [48]. Interestingly, we also found an increased *BATF3* expression in sDCs derived from LD-infected mice. Accordingly, one may expect that this increased *BATF3* expression will promote cDC1 predominance, leading to disease protection. However, due to the reduced expression of *IRF8* (another transcription factor necessary for DC differentiation to cDC1), BATF3 alone could not increase the cDC1 abundance in LD-infected mice [32, 33]. Rather, our immunophenotypic data depicted cDC2 as a more abundant DC subtype over cDC1 in these infected mice. A possible reason for the low abundance of cDC1 could be due to IL-10, which is produced at an elevated level by various cells (e.g., DCs [our current findings], T cells [27], macrophages [49], etc.) during LD infection. This proposition is based on our finding that incubation of spleen cells with IL-10 lessens cDC1 abundance (Supplementary Fig. 11). Our result is consistent with a previous report demonstrating that IL-10 produced by tumor-associated macrophages type-2 (TAM2) reduces the number of CD103-expressing DCs [50]. Notably, CD103 is a surface marker of cDC1 [25, 51, 52]. Altogether, our findings point to the emergence of cDC2 as a major DC subtype during chronic LD infection. Our data also suggest that TIM-3 acts as a critical receptor in driving this cDC2 bias. This was supported by the observation that the adoptive transfer of TIM-3-silenced BMDCs prevented the predominance of cDC2 in chronically infected mice. Interestingly, we noted that TIM-3 expression was upregulated on both cDC1 and cDC2 following LD infection and that this upregulation was more pronounced on cDC1 than on cDC2. One possible explanation for higher TIM-3 expression on cDC1 during LD infection is that TIM-3, being an inhibitory receptor [16], might be restraining cDC1 from exhibiting proinflammatory effects via STAT3. The latter was found to be highly activated in cDC1 during chronic LD infection and is reported to impede cDC1 activity [53]. Additionally, the increased TIM-3 level on cDC1 might in part have contributed to the reduction of the cDC1 population in LD-infected mice by promoting IL-10 secretion. In this regard, we have discussed below how TIM-3 promotes IL-10 secretion from DCs in response to LD infection. Nevertheless, our data suggest that LD upregulates TIM-3 expression on DCs regardless of the DC subtype. However, among these DC subtypes, TIM-3 was majorly expressed by cDC2 (although TIM-3 expression level on cDC2 was lower than that on cDC1) in LD-infected mice. In contrast, only a few cDC1 (being a low abundant population under infected conditions) expressed TIM-3. Overall, these observations demonstrate a previously unrecognized ability of LD to influence the splenic cDC1-cDC2 balance by regulating TIM-3 expression on DCs.

We further checked whether TIM-3 or any other receptor contributed to LD-mediated TIM-3 upregulation on DCs. For this, we blocked TIM-3 with anti-TIM-3 antibody and verified whether it affected LD-induced TIM-3 upregulation on DCs. This approach allowed other receptors to interact freely with LD while TIM-3 was blocked on the DC surface. However, we still noted the inhibition of LD-induced TIM-3 upregulation on DCs upon anti-TIM-3 antibody treatment. This finding ruled out the involvement of other receptors (or receptors) in LD-mediated TIM-3 upregulation on DCs and established TIM-3’s critical role in this process. Of note, we have recently demonstrated that LD directly activates TIM-3 signaling (as measured by TIM-3 phosphorylation) in DCs [24], although the identity of TIM-3-interacting LD surface antigen (or antigens) is yet to be determined. Furthermore, LD induces this TIM-3 phosphorylation within 2.5 min after incubation [24]. Given these observations, it is unlikely that other receptors were activated prior to TIM-3 induction by LD. Therefore, LD-mediated induction of TIM-3 can be considered as the initial stimulus for TIM-3 upregulation and the successive DC polarization to the cDC2 subtype during chronic LD infection.

Second, our results have shown that LD promotes TIM-3 upregulation on DCs and subsequent cDC2 bias in a STAT3-dependent manner. Previously, few studies have reported that STAT3 is mildly induced in macrophages upon LD infection [54, 55]. Our data demonstrated that LD infection triggered STAT3 activation also in DCs and that LD mediated this effect through TIM-3. The activation of STAT3 in turn promoted TIM-3 upregulation on DCs. Recently, the blockade of STAT3 using pharmacological inhibitors has been shown to decrease TIM-3 expression in regulatory T cells [56]. However, it is currently unknown whether STAT3 plays any role in regulating TIM-3 expression in DCs or any other cells. In addition, the molecular mechanism by which STAT3 mediates such regulatory effect remains undefined. Here we showed that LD-mediated STAT3 induction promoted TIM-3 upregulation on DCs by triggering IL-10 secretion. For instance, silencing of STAT3 effectively blocked LD-stimulated IL-10 secretion from DCs. In addition, neutralization of IL-10 with anti-IL-10 antibody prevented LD-induced upregulation of TIM-3 expression on DCs. Although STAT3 was earlier proposed to mediate IL-10 expression [39], its exact binding site in the murine *IL-10* promoter is still unclear. Furthermore, the role of STAT3 in LD-induced IL-10 production has never been experimentally validated. Our study has identified a STAT3-binding site in the murine *IL-10* promoter and demonstrated its pivotal role in driving the *IL-10* promoter activity. Together, these results provide the molecular basis for LD-induced IL-10 production mediated by STAT3 in DCs. Our findings have also deciphered the mechanism by which STAT3-driven IL-10 production upregulated TIM-3 expression on DCs following LD infection. We have demonstrated that STAT3-mediated IL-10 production during LD infection augmented c-Src activation, which then recruited Ets and USF transcription factors to the *HAVCR2* promoter and thereby increased TIM-3 expression on DCs. This result is consistent with our earlier report [37] demonstrating a key role for c-Src-Ets, USF signaling in recombinant IL-10-mediated TIM-3 upregulation on DCs. Thus, we have identified STAT3→IL-10→c-Src→Ets, USF as a key pathway driving LD-induced TIM-3 upregulation on DCs. Our subsequent investigation established that STAT3-driven TIM-3 upregulation plays a pivotal role in promoting cDC2 dominance in chronically infected mice. We have proven this fact in vivo by adoptive transfer experiments, in which the repeated transfer of STAT3-silenced BMDCs into chronically infected mice reduced cDC2 prevalence and simultaneously augmented the cDC1 abundance. However, TIM-3 overexpression in STAT3-silenced BMDCs blocked these effects. Strikingly, we noted more STAT3 activation in cDC1 than in cDC2 in mice with chronic LD infection. The latter observation correlates with much higher TIM-3 expression on cDC1 than cDC2 during the chronic phase of LD infection. Aside from upregulating TIM-3 expression, the increased STAT3 activation was possibly needed to attenuate the proinflammatory activity of cDC1 in LD-infected mice by promoting IL-10 secretion. This possibility has been spurred by a recent study showing that the anti-inflammatory signaling molecule STAT3 plays an essential role in IL-10-mediated suppression of cDC1 activity [53]. Given this and other reports [25, 50–52] coupled with our observation demonstrating STAT3 as a key factor for LD-induced IL-10 production by DCs, it is likely that the reduced IL-10 production by STAT3-silenced BMDCs might play a role in increasing cDC1 population in chronically infected mice following adoptive transfer. Notably, the role of STAT3 in regulating the cDC1-cDC2 balance has remained unrecognized so far. Our findings, therefore, depict a new role for the STAT3-TIM-3 axis in determining the DC subtype bias.

The third key observation made in this study is that STAT3-TIM-3 pathway-mediated cDC2 abundance promotes Th2 bias and thus contributes to disease pathogenesis during LD infection. This was evident by our observations that the adoptive transfer of STAT3-silenced BMDCs markedly reduced Th2 response, hepatosplenomegaly, and the liver and splenic parasite burden in LD-infected mice. Such inhibitions were greatly minimized when we transferred STAT3-silenced BMDCs after TIM-3 overexpression. Our findings are in agreement with the notion that splenic cDC2 promotes Th2 responses [20]. However, whether cDC2 subtype dominance, mediated by the STAT3-TIM-3 axis, is responsible for increased Th2 response and disease pathogenesis during LD infection was not known. A previous study has suggested that STAT3 acts as a disease-exacerbating factor [57]. However, it remains ill-defined how STAT3 mediates this disease-promoting activity. Likewise, although TIM-3 has been reported to negatively regulate Th1 immunity [58], it is still unknown whether TIM-3 mediates this effect by regulating cDC1-cDC2 balance. An understanding of all these issues is critically important in view of the fact that the balance between Th1 and Th2 immunity determines the disease outcome during LD infection [42–44]. In this regard, our above-mentioned observations have revealed the mechanism that STAT3-driven TIM-3 upregulation on DCs contributes to disease pathogenesis by promoting cDC2 abundance and subsequently favoring Th2 response during LD infection.

Now, one may wonder what could be the upstream activator of STAT3. This is because STAT3 is not yet known to be directly activated by TIM-3. In this respect, we have recently demonstrated that upon LD-mediated induction, TIM-3 recruits and activates Btk [24]. After that, Btk promotes IL-10 production by DCs [24]. Our current findings have shown that Btk mediated LD-stimulated IL-10 production by activating STAT3 and subsequently facilitating STAT3 binding to the *IL-10* promoter. Ultimately, Btk promoted LD-induced TIM-3 upregulation on DCs. Thus, Btk serves as a signal conveyor from LD-induced TIM-3 to STAT3.

**Fig. 8:**
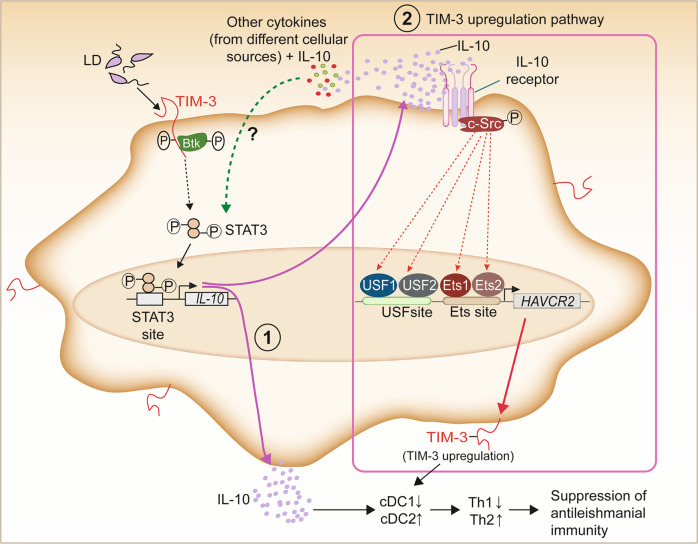
Model illustrating how DC-expressed TIM-3 promotes cDC2 prevalence and disease-exacerbating Th2 response in LD-infected mice. Our results have demonstrated that the in vivo cDC1-cDC2 balance gets shifted toward the cDC2 subtype during chronic LD infection and that TIM-3 expressed by DCs plays a critical role in mediating this process. TIM-3 promotes this cDC2 dominance (and reduces cDC1 abundance) in LD-infected mice by enhancing IL-10 production via the Btk-STAT3 signaling pathway [marked as (1)]. The latter pathway is triggered upon LD-mediated TIM-3 activation (ref. [24] and our current findings). In vivo, it is also possible that IL-10, together with other cytokines secreted from various cellular sources, reinforces its production by activating STAT3. Further investigation, however, is needed to confirm this hypothesis. Nevertheless, the IL-10 so produced also activates the c-Src→Ets, USF pathway to upregulate TIM-3 expression on DCs [marked as (2)]. The enhanced TIM-3 expression further contributes to increased cDC2 abundance and decreased cDC1 population in LD-infected mice. The predominance of the cDC2 subtype then favors the development of Th2 responses that promote disease pathogenesis.

In summary, the present study provides evidence for a cDC2-dominated immune response that contributes to disease pathogenesis during LD infection and has demonstrated that TIM-3 expressed by DCs plays a pivotal role in driving this process. In response to LD infection, TIM-3 triggers STAT3 activation through Btk and thus promotes IL-10 production in DCs. Although we have shown in this study that IL-10 does not activate STAT3 in DCs during LD infection, in an in vivo setting, it remains possible that IL-10, along with other cytokines secreted by different cell types, can activate STAT3 and increase its own production. Confirmation of the latter possibility, however, requires further investigation. Nevertheless, our findings demonstrate that LD induces STAT3 activation and subsequent IL-10 production in DCs via TIM-3. Importantly, both STAT3 and IL-10 are reported to reduce the cDC1 cell numbers [50, 53]. This possibly favors the predominance of cDC2 in chronically LD-infected mice. Our results have also shown that TIM-3 expression is upregulated on DCs following LD infection and that LD mediates this effect by activating a TIM-3 signaling pathway Btk→STAT3→IL-10→c-Src→Ets1/2, USF1/2. The elevated TIM-3 expression on DCs then aids in decreasing the cDC1 population and increasing cDC2 abundance in chronically infected mice. Eventually, the increased cDC2 abundance leads to the augmentation of disease-promoting Th2 responses and subsequent disease pathology during LD infection (Fig. 8). Finally, we have shown that suppression of STAT3-driven TIM-3 expression in DCs reduces cDC2 abundance and successive Th2 response, and thereby confers protection against LD infection. Accordingly, targeting STAT3 with clinically accessible STAT3 inhibitors such as atovaquone or WP1066 [59, 60] is expected to reduce TIM-3 expression and the cDC2 population in LD-infected hosts while increasing cDC1 abundance. Alternatively, the cDC1 expansion and activation may be significantly improved by the combined administration of FLT3 ligand (FLT3L) and poly (I:C) [61, 62]. Although further investigation is needed to determine the efficacy of FLT3L/poly (I:C) combination therapy or the above-mentioned STAT3 inhibitors in raising cDC1 abundance, our data support the idea that increasing the cDC1 predominance might be a promising strategy for immunotherapy of VL. Overall, our study has deciphered a unique immunoregulatory mechanism promoting disease pathology during LD infection and identified a key role for TIM-3 in mediating this process.

## Materials and methods

### Reagents

Following antibodies were used for immunoblot assays: anti-STAT3 (9139), anti-phospho-STAT3 (Tyr705, 9131), anti-TIM-3 (83882S), anti-Btk (8547S), anti-phospho-Btk (Tyr223, 5082S), anti-c-Src (2109), and anti-phospho-c-Src (Tyr416, 2101; all were from Cell Signaling Technologies, MA, USA); anti-Ets1 (sc-350), anti-Ets2 (sc-351), anti-USF1 (sc-229), anti-USF2 (sc-862), HRP-labeled anti-rat IgG (sc-2032), and anti-β-actin (sc-47778; all were from Santa Cruz Biotechnology, Texas, USA); and anti-IL-10 (MAB417), HRP-labeled anti-rabbit IgG (HAF008), and anti-mouse IgG (HAF018; all from R & D system, Minneapolis, MN, USA). For the ChIP assay, the antibodies and reagents used are mentioned below: anti-STAT3 (4904; Cell Signaling Technologies), rabbit IgG (2729; Cell Signaling Technologies), and protein A/G PLUS-Agarose beads (sc-2003: Santa Cruz Biotechnology). Following antibodies from BioLegend (CA, USA) were used for flow cytometry analyses: FITC-labeled anti-mouse CD11c (117306), APC-labeled anti-mouse CD11c (117310), APC/Cyanine7-labeled anti-mouse/human CD11b (101226), PE-labeled anti-mouse CD8α (100708), PE-labeled anti-mouse TIM-3 (134004; clone B8.2C12), APC-labeled anti-mouse TIM-3 (134008; clone B8.2C12), PE/Cyanine7-labeled anti-mouse CD3 (100220), PerCP/Cyanine5.5-labeled anti-mouse CD4 (100434), APC-labeled anti-mouse IL-10 (505010), and FITC-labeled anti-mouse IFN-γ (505806). Isotype control antibodies such as PE-labeled rat IgG1,κ (400408), APC-labeled rat IgG1,κ (400412), APC-labeled rat IgG2b,κ (400611), and FITC-labeled rat IgG1,κ (400405) were also obtained from BioLegend. Other isotype controls, such as mouse IgG2a (61656) and rabbit IgG (2729), used for flow cytometry analysis, were procured from Cell Signaling Technologies. Neutralizing anti-IL-10 (505002; clone JES5-16E3) and rat IgG2b,κ (400643; isotype control antibody) were obtained from BioLegend (CA, USA). The anti-TIM-3 monoclonal antibody (BE0115, clone RMT3-23) and corresponding isotype control rat IgG2a,κ (BE0089) used for in vitro TIM-3 blocking experiments were purchased from Bio X cell (Lebanon, NH, USA) [24]. Recombinant mouse granulocyte-macrophage colony-stimulating factor (GM-CSF) and IL-4 were procured from Peprotech Asia (Rehovot, Israel), and recombinant IL-10 was purchased from BioLegend. The ON-TARGETplus non-targeting control pool siRNAs and SMARTpool siRNAs targeting mouse TIM-3, Btk, STAT3, IL-10, c-Src, Ets1, Ets2, USF1, and USF2 were purchased from Dharmacon (CO, USA). Anti-mouse CD11c magnetic microbeads were procured from Miltenyi Biotec (Germany). Dynabeads M-280 Streptavidin used for DNA pull-down assay was purchased from Thermo Fisher Scientific (MA, USA). All other reagents were obtained from Sigma-Aldrich (India).

### Animals

BALB/c mice and golden hamsters (*Mesocricetus auratus*) were maintained and bred under pathogen-free conditions at IMTECH Centre of Animal Resources and Experimentation (iCARE) of the CSIR-Institute of Microbial Technology.

### Parasite

The LD strain AG83 [MHOM/IN/83/AG83; American Type Culture Collection (ATCC PRA-413); gifted by Dr. Nahid Ali (CSIR-Indian Institute of Chemical Biology, India)] was maintained in golden hamsters [3, 63]. Amastigotes were isolated from the spleens of infected hamsters and then transformed and maintained as promastigotes, as described earlier [64, 65].

### DC preparation and LD infection

BMDCs were prepared from male or female BALB/c mice (8–12 weeks old) as described [66]. BMDCs (5 × 10^6^ cells/well) were then infected in vitro with LDPm (stationary phase) or LDAm at parasite to DC ratio of 10:1 for specified times in RPMI 1640 complete media (10% FBS, L-glutamine, non-essential amino acids, sodium pyruvate, penicillin/streptomycin, and 2-mercaptoethanol). In some experiments, BMDCs were infected with LDPm or LDAm in the presence or absence of 10 μg/ml neutralizing anti-IL-10 antibody or rat IgG2b,κ (isotype-matched control antibody). In some other experiments, we treated BMDCs with a TIM-3 blocking antibody (clone RMT3-23) or isotype control antibody before incubation with LDPm, as described earlier [24].

### Quantitative RT-PCR

The cDNA synthesis and quantitative RT-PCR analyses were carried out using the Go Taq 1-step RT-qPCR System (Promega, WI, USA) and the primers specific for the following mouse genes: *IRF4*, forward 5’-AGATTCCAGGTGACTCTGTG-3’ and reverse 5’-CTGCCCTGTCAGAGTATTTC-3’; *KLF4*, forward 5’-GCGAACTCACACAGGCGAGAAACC-3’ and reverse 5’-TCGCTTCCTCTTCCTCCGACACA-3’; *IRF8*, forward 5’-GGATATGCCGCCTATGACACA-3’ and reverse 5’-CATCCGGCCCATACAACTTAG-3’; *BATF3*, forward 5’-CGGAAGAAGCAGACCCAGAA-3’ and reverse 5’-GGTGACGCAGCTCCTCCTT-3’; *HAVCR2*, forward 5’-TTGGAGTGGGAGTCTCTGCT-3’ and reverse 5’-AATCCTGACTGCTCCTGCAT-3’; and *ACTB* (encoding β-actin), forward 5′-GCTCTGGCTCCTAGCACCAT-3′ and reverse 5′-GCCACCGATCCACACCGCGT-3′. Gene expression was measured by the change in threshold method (ΔΔC_T_) and normalized to the *ACTB* mRNA expression.

### EMSA, DNA pull-down, and immunoblot analysis

Nuclear extracts were prepared as described [67]. EMSA was performed as described [68] using various P^32^-labeled DNA probes (Supplementary Table 1) specific for mouse *IL-10* or *HAVCR2* promoter. Bands were visualized using a phosphoimager (PharosFX Molecular Imager, Bio-Rad). Immunoblot analysis was carried out as described [66]. The pull-down of DC nuclear proteins using streptavidin (SA)-coupled Dynabeads and indicated biotinylated oligonucleotides (see Supplementary Table 1) followed by immunoblot analysis was performed as illustrated previously [69]. Densitometry quantification was done using Scion Image software (Scion Corporation, Maryland, USA).

### ChIP

ChIP assay was carried out with rabbit IgG or anti-STAT3 antibody using the ChIP-IT kit (Active Motif, CA, USA) following the manufacturer’s instructions. Precipitated DNA was analyzed by quantitative PCR using mouse *IL-10* promoter-specific primers mentioned in Supplementary Table 1. Results were normalized to ChIP with rabbit IgG (nonspecific background) and input DNA.

### Reporter assay

The pGL2-luciferase reporter plasmid containing wild-type mouse *IL-10* promoter fragment [−1541 to −1 region; *IL-10* (Wt) pro] was procured from Addgene (MA, USA). Subsequently, this promoter fragment was cloned into a pGL3-luciferase reporter plasmid (Promega) using XhoI and HindIII sites. A similar promoter fragment containing a mutated STAT3-binding site [*IL-10* (Mut) pro], cloned in the pGL3-luciferase reporter vector, was synthesized from Biomatik (Ontario, Canada). HEK293T cells (2.5 × 10^5^ cells/well) were transfected with either of these reporter constructs (400 ng) together with 400 ng mammalian expression vector (pBabe) expressing mouse STAT3 or corresponding empty vector (gifted by Prof. Albert Baldwin, University of North Carolina at Chapel Hill, North Carolina, USA) and 200 ng *Renilla* luciferase reporter plasmid (pRL-CMV, an internal control; Promega) using the FreeStyle MAX Reagent (Thermo Fisher Scientific). At 24 h following transfection, luciferase activity was measured with the dual-luciferase reporter assay kit (Promega). HEK293T cells (CRL-3216; ATCC) were obtained as a gift from Dr. Ashwani Kumar (CSIR-Institute of Microbial Technology, Chandigarh, India).

### RNA-mediated interference

DCs were transfected with 60 nM siRNA using Lipofectamine RNAiMAX reagent (Thermo Fisher Scientific).

### Assessment of splenic cDC1 and cDC2 abundance, and analysis of TIM-3 expression on cDC1, cDC2, and total sDCs in LD-infected mice

BALB/c mice (4–6 weeks old) were infected intravenously with stationary phase LDPm (1 × 10^7^ parasites/mouse) or left uninfected. On the 45th day postinfection, splenocytes were prepared from these LD-infected mice or age-matched uninfected mice. The abundance of cDC1 and cDC2 subtypes in splenocytes was determined by measuring the percentage and number of CD11b^−^CD8α^+^ and CD11b^+^CD8α^−^ cells within the CD11c-gated population, respectively, by flow cytometry. In addition, the frequency and number of TIM-3-expressing cDC1 (CD11c^+^CD11b^−^CD8α^+^) and cDC2 (CD11c^+^CD11b^+^CD8α^−^) and the level of TIM-3 expression on these DC subtypes were assessed via flow cytometry. In some experiments, the expression levels of TIM-3 on sDCs from uninfected and LD-infected mice were compared. For this purpose, mice were either infected with LD or left uninfected, and splenocytes were prepared on various days after infection. The expression of TIM-3 on sDCs (CD11c-gated population) was analyzed by flow cytometry.

### Analysis of phospho-STAT3 and total STAT3 expression in cDC1 and cDC2

Splenocytes from uninfected and 45-day-infected mice were first surface-stained with anti-CD11c-FITC, anti-CD8α-PE, and anti-CD11b-APC/Cyanine7. Splenocytes were then immunostained intracellularly with anti-phospho-STAT3 antibody or rabbit IgG, followed by anti-rabbit IgG-Alexa Fluor 633 (A-21070; Thermo Fisher Scientific) using a Fixation/Permeabilization Buffer kit (88-8823-88; Thermo Fisher Scientific). Alternatively, immunostaining was done with anti-STAT3 antibody or mouse IgG2a followed by anti-mouse IgG-Alexa Fluor 633 (A-21063; Thermo Fisher Scientific). The expression of phospho-STAT3 and total STAT3 in cDC1 and cDC2 subtypes was evaluated by flow cytometry. The procedure followed for the identification of cDC1 and cDC2 populations is mentioned above.

### Adoptive transfer of DCs

BALB/c BMDCs (1 × 10^6^) were transfected (or not) with control siRNA or TIM-3-specific siRNAs. DCs (1 × 10^6^) were then transferred intravenously into LD-infected BALB/c mice (on days 15, 25, 35, and 45 postinfection). In some cases, both LD-infected mice and age-matched uninfected mice were left without any DC transfer. On the 48th day after LD infection, spleens were removed from these mice, and splenocytes were analyzed to determine the frequency of cDC1 and cDC2 populations as described above.

For another DC adoptive transfer experiment, BALB/c BMDCs (1 × 10^6^) were transfected with STAT3-specific siRNA or control siRNA. In some experimental sets, STAT3 siRNA-treated BMDCs were transfected with 1 μg empty vector or TIM-3-expressing vector (BALB/c TIM-3/pMKITneo; gifted by Prof. H. Akiba, Juntendo University, Tokyo, Japan) using the FreeStyle MAX Reagent (Thermo Fisher Scientific). DCs (1 × 10^6^) were then transferred into LD-infected BALB/c mice on days 15, 25, 35, and 45 postinfection. On day 48 postinfection, spleens and livers were isolated to measure their weights and parasite load and to determine the frequency of cDC1 or cDC2 subtypes and IFNγ- or IL-10-producing CD4^+^ and CD8^+^ T cells in the spleens. The parasite burden in the spleen and liver was measured by the stamp-smear method and is presented as Leishman-Donovan Unit (LDU) [70]. The frequency of the cDC1 or cDC2 population was assessed as mentioned above. For analyzing the expression of IFNγ and IL-10 within CD4^+^ and CD8^+^ T cells, splenocytes were first surface-stained with anti-CD3-PE/Cyanine7 along with anti-CD4-PerCP/Cyanine5.5 or anti-CD8-PE. Afterward, splenocytes were undergone intracellular immunostaining using a Fixation/Permeabilization Buffer kit (mentioned above), and FITC-labeled anti-mouse IFNγ, APC-labeled anti-mouse IL-10, or FITC/APC-labeled rat IgG (isotype control). The percentage of IFNγ- or IL-10-expressing CD4^+^ and CD8^+^ T lymphocytes within the CD3-gated population was measured by flow cytometry.

To determine the frequency and time span that the adoptively transferred DCs stayed in the spleens of recipient mice, BALB/c BMDCs were labeled with eFluor 670 dye (5 μM; eBioscience, San Diego, CA, USA) and then transferred intravenously into uninfected syngeneic mice. On days 1, 3, and 7 post-DC transfer, splenocytes were isolated from recipient mice and immunostained with anti-CD11c antibody. The frequency of eFluor 670^+^ cells within the CD11c-gated population (i.e., transferred DCs) was measured via flow cytometry. In addition, to assess the efficiency of TIM-3 or STAT3 knockdown in BMDCs localized to the spleens of recipient mice after adoptive transfer, BMDCs were either left untransfected or transfected with control siRNA, STAT3-specific siRNA, or TIM-3-specific siRNA before labeling with eFluor 670 dye. BMDCs were then adoptively transferred into uninfected syngeneic mice as described above. After one day, splenocytes from these mice were subjected to intracellular immunostaining for STAT3 (using PE-labeled anti-STAT3, 678007; Biolegend) or surface staining for TIM-3 (using PE-labeled anti-TIM-3, 134004; Biolegend). In both cases, cells were surface-stained for CD11c as well. The STAT3 and TIM-3 knockdown efficiency in adoptively transferred DCs (i.e., eFluor 670^+^ cells within CD11c-gated population) was assessed via flow cytometry and calculated as follows:

$$\frac{{{{{\mathrm{\% }}}}\;{{{\mathrm{eFluor}}}}\;670^ + {{{\mathrm{STAT}}}}3^ + {{{\mathrm{DC}}}}\left( {{{{\mathrm{control}}}}\;{{{\mathrm{siRNA}}}} - {{{\mathrm{transfected}}}}} \right) - {{{\mathrm{\% }}}}\;{{{\mathrm{eFluor}}}}\;670^ + {{{\mathrm{STAT}}}}3^ + {{{\mathrm{DC}}}}({{{\mathrm{STAT}}}}3\;{{{\mathrm{siRNA}}}} - {{{\mathrm{transfected}}}})}}{{{{{\mathrm{\% }}}}\;{{{\mathrm{eFluor}}}}\;670^ + {{{\mathrm{STAT}}}}3^ + {{{\mathrm{DC}}}}({{{\mathrm{control}}}}\;{{{\mathrm{siRNA}}}} - {{{\mathrm{transfected}}}})}} \times 100$$

and

$$\frac{{{{{\mathrm{\% }}}}\;{{{\mathrm{eFluor}}}}\;670^ + {{{\mathrm{TIM}}}} - 3^ + {{{\mathrm{DC}}}}\left( {{{{\mathrm{control}}}}\;{{{\mathrm{siRNA}}}} - {{{\mathrm{transfected}}}}} \right) - {{{\mathrm{\% }}}}\;{{{\mathrm{eFluor}}}}\;670^ + {{{\mathrm{TIM}}}} - 3^ + {{{\mathrm{DC}}}}({{{\mathrm{TIM}}}} - 3\;{{{\mathrm{siRNA}}}} - {{{\mathrm{transfected}}}})}}{{{{{\mathrm{\% }}}}\;{{{\mathrm{eFluor}}}}\;670^ + {{{\mathrm{TIM}}}} - 3^ + {{{\mathrm{DC}}}}({{{\mathrm{control}}}}\;{{{\mathrm{siRNA}}}} - {{{\mathrm{transfected}}}})}} \times 100$$

### Flow cytometry

Flow cytometry was carried out with a FACSVerse flow cytometer (BD Biosciences). Data were analyzed with FlowJo software (Tree Star).

### Assessment of IL-10 production by DCs

BMDCs (1 × 10^6^ cells/ml) were infected with LDPm or LDAm for indicated times as described above. In some experiments, BMDCs were transfected with control siRNA or siRNA specific for TIM-3 or STAT3 before LD infection. The culture supernatants were assayed for IL-10 using an ELISA kit (BioLegend) following the manufacturer’s instructions.

### Analysis of cDC1 and cDC2 population after IL-10 treatment

Splenocytes (1 × 10^6^ cells/well plated with 1 ml RPMI 1640 complete medium in a 24-well low-cluster plate) from uninfected mice were treated with recombinant IL-10 (250 ng/ml) for 72 h. The frequency and number of cDC1 and cDC2 were determined via flow cytometry, as illustrated above.

### Statistical analysis

One-way ANOVA (SigmaPlot 11.0 program) was used for statistical analyses. A *p*-value <0.05 was considered significant.

## Supplementary information

Supplemental Material

CDD Checklist

## Data Availability

All data generated during this study are included either in the main article or in the supplementary information files.
