# Supplementary material for: TIM-3 increases the abundance of type-2 dendritic cells during Leishmania donovani infection by enhancing IL-10 production via STAT3
Source: Cell Death Dis. 2023 May 18;14(5):331. doi: 10.1038/s41419-023-05848-3 (PMC10195822; doi:10.1038/s41419-023-05848-3)
Supplement: Supplementary file 1 — Supplemental Material [file 41419_2023_5848_MOESM1_ESM.docx]

**Supplementary Information**

**Supplementary Table 1.** **Details of primers and oligonucleotides used in this study**

Primer sequences for ChIP-qPCR analyses of the mouse *IL-10* promoter

| Promoter | Regions amplified | Forward primer sequence (5'→3'*)* | Reverse primer sequence  (5'→3') |
| --- | --- | --- | --- |
| Mouse *IL-10* | -194 to +37 | GCAGAAGTTCATTCCGACCA | TGTGGCTTTGGTAGTGCAAG |

All position numbers are relative to the transcription start sites.

Oligonucleotides used for EMSA and DNA pull-down assay

| Probes | Positions | Sequence (5'→3') |
| --- | --- | --- |
| **Mouse *IL-10* promoter** |  |  |
| i) Pr (carrying wild-type  STAT site) | -68 to -38 | CAAAAACCTTTGCCAGGAAGGCCCCACTGAG |
| ii) Mut-Pr (carrying  mutant STAT site) | -68 to -38 | CAAAAACCTTTGCCA*AATC*GGCCCCACTGAG |
|  |  |  |
| **Mouse *HAVCR2* promoter** |  |  |
| i) Ets-Pr (carrying wild-  type Ets site) | -109 to -85 | TCACTGGAGGTCAGACATCCTGGGG |
| ii) MutEts-Pr (carrying  mutant Runx site) | -109 to -85 | TCA*A*T*AA*A*AA*TCAGACATCCTGGGG |
| iii) USF-Pr (carrying  wild-type USF site) | -125 to -108 | AGTACTAACGTGGTAATC |
| iv) MutUSF-Pr (carrying  mutant USF site) | -125 to -108 | AGTACTAA*TTAA*GTAATC |

All position numbers are relative to the transcription start sites; binding sites for the transcription factors are underlined, and mutated bases are italicized.

**Supplementary Data**

**
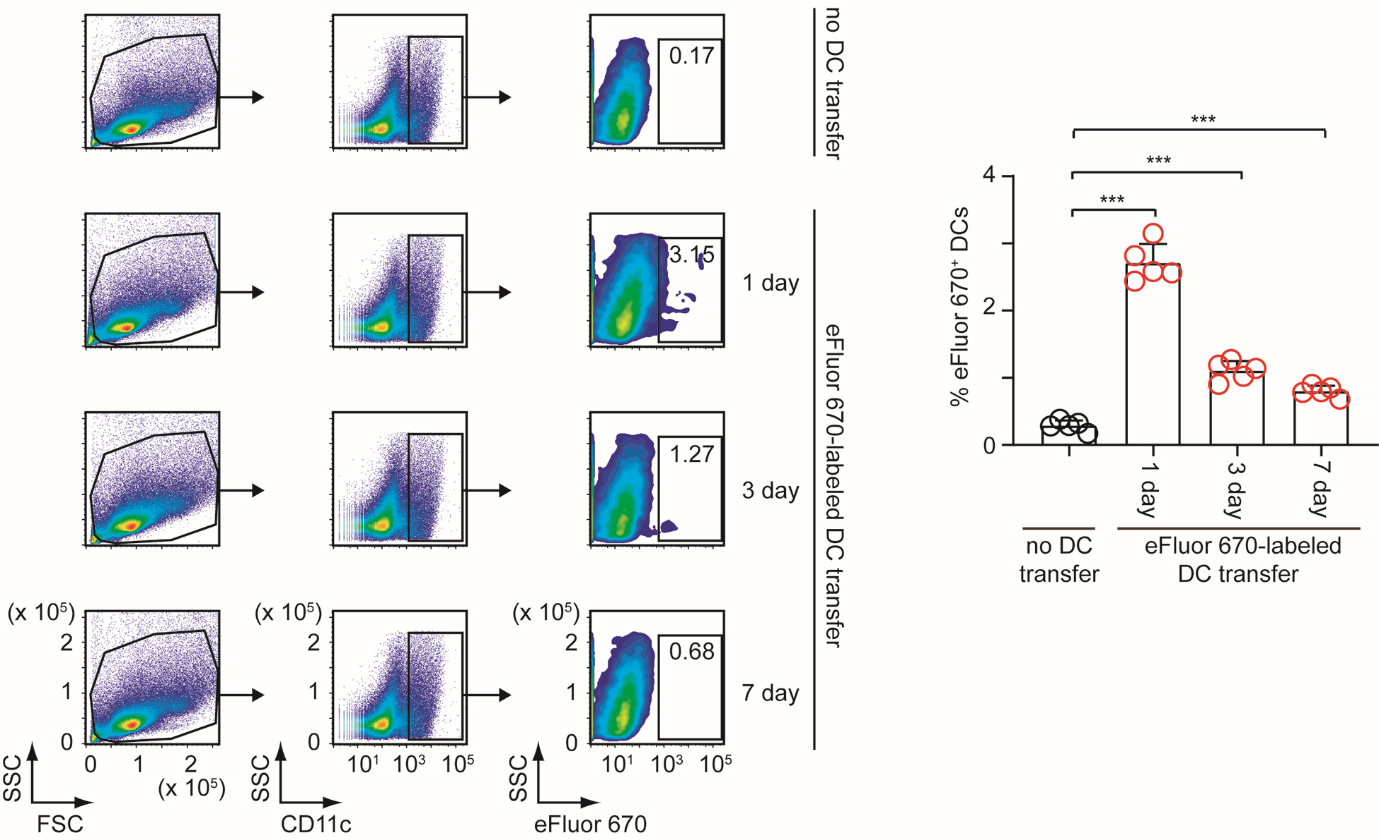
**

**Supplementary Fig. 1 Detection of adoptively transferred DCs in the spleens of recipient mice.** BALB/c BMDCs were labeled with eFluor 670 dye and then adoptively transferred into uninfected syngeneic mice. On days 1, 3, and 7 post-DC transfer, splenocytes from recipient mice were immunostained with anti-CD11c antibody. The frequency of eFluor 670^+^ cells within CD11c-gated population (i.e., transferred DCs) was measured via flow cytometry (representative data of *n* = 5; left). Right, the bar graph shows compiled data (*n* = 5 mice per group). Error bars indicate SD. ^***^*p* < 0.001.

**
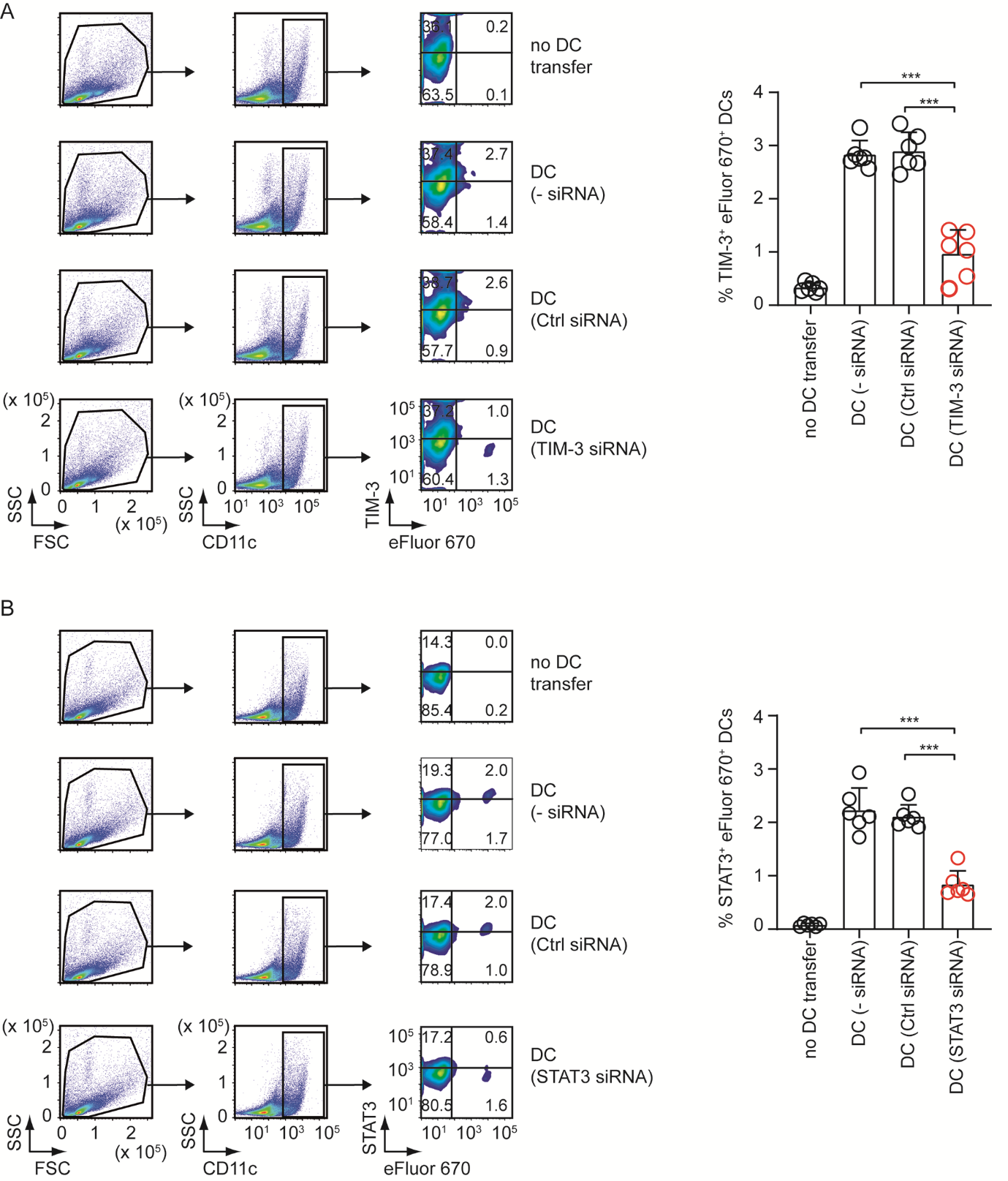
**

**Supplementary Fig. 2 TIM-3 and STAT3 knockdown efficiency in adoptively transferred DCs localized in recipient mice spleens.** BALB/c BMDCs were transfected with indicated siRNAs or left untransfected, labeled with eFluor 670 dye, and then adoptively transferred into uninfected syngeneic mice. After one day, splenocytes were prepared from recipient mice and immunostained for CD11c together with TIM-3 (**A**) or STAT3 (**B**). The TIM-3 and STAT3 knockdown efficiency in adoptively transferred BMDCs (i.e., eFluor 670^+^ cells within CD11c-gated population) was evaluated by flow cytometry (see “Materials and methods” for further details). The left panel shows representative data out of *n* = 6, and the right panel (bar graphs) depicts compiled data (*n* = 6 mice per group). Error bars indicate SD. ^***^*p* < 0.001.

**
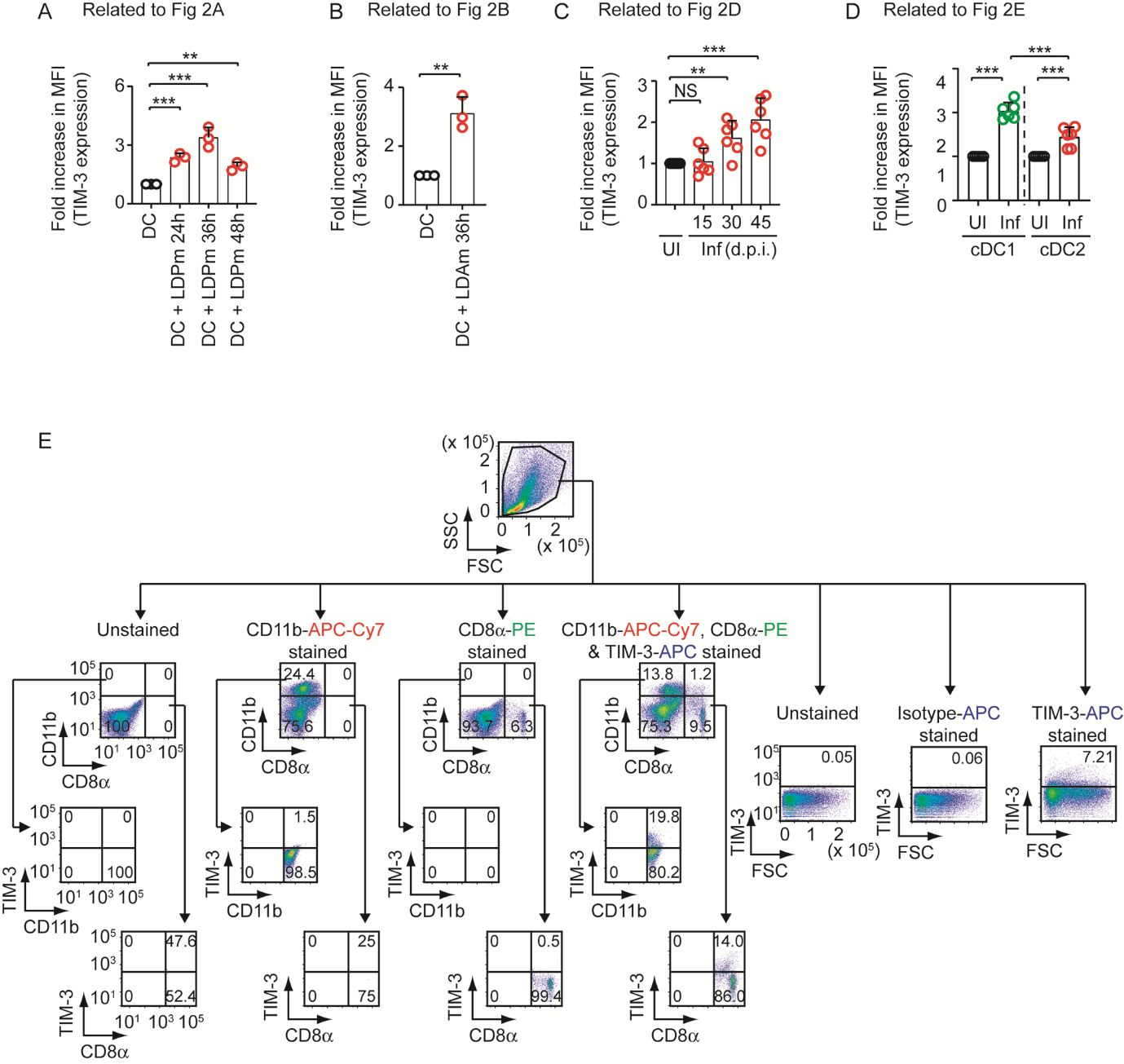
**

**Supplementary Fig. 3 Supporting information for Fig. 2.** **A, B** Related to Fig. 2A and B. BMDCs were either left uninfected (DC) or infected with LDPm (**A**; relates to Fig. 2A) or LDAm (**B**; relates to Fig. 2B) for indicated times. Flow cytometry analysis depicting TIM-3 expression on DCs has been shown in Fig. 2A and B. The mean fluorescence intensity (MFI) of corresponding TIM-3 expression was measured after subtracting the background signal (isotype control) and is presented here as fold change relative to uninfected DCs. Compiled data of *n* = 3 are presented here. Each symbol represents data of individual replicate. **C** Related to Fig. 2D. Bar graphs showing compiled data of *n* = 6 mice per group for MFI of TIM-3 expression on sDCs (i.e., CD11c-gated cells) derived from uninfected (UI) or day 15, 30, or 45 infected (Inf) mice. The MFI values were calculated as in panel **A** and presented as fold change compared to that of sDCs derived from uninfected mice. The corresponding flow cytometry data have been shown in Fig. 2D. Each symbol corresponds to the data of an individual mouse. d.p.i, days postinfection. **D** Related to Fig. 2E. The MFI of TIM-3 expression on cDC1 and cDC2 population in uninfected and 45-day-infected mice (flow cytometry data have been presented in Fig. 2E) was measured as in panel **A** and presented as fold change relative to uninfected mice. The bar diagram depicts the compiled data of *n* = 6 mice per group. Each symbol denotes the data of an individual mouse. **E** Gating strategy for TIM-3 expression (based on single-stained controls) related to Fig. 2E. Total splenocytes were first gated based on forward, and side scatter. Then, the gate for TIM-3^+^ cells within the CD8α^+^ or CD11b^+^ population was set based on unstained and single-stained controls. The accuracy of this TIM-3 gating strategy was further ascertained by applying the same gate in unstained, isotype control-stained, and anti-TIM-3 antibody-stained splenocytes to analyze the TIM-3^+^ population. Notably, the same gate was also used to analyze the TIM-3^+^ population for experiments mentioned in Fig. 2E. Error bars represent SD. ^**^*p* < 0.01, ^***^*p* < 0.001; NS, not significant.

**
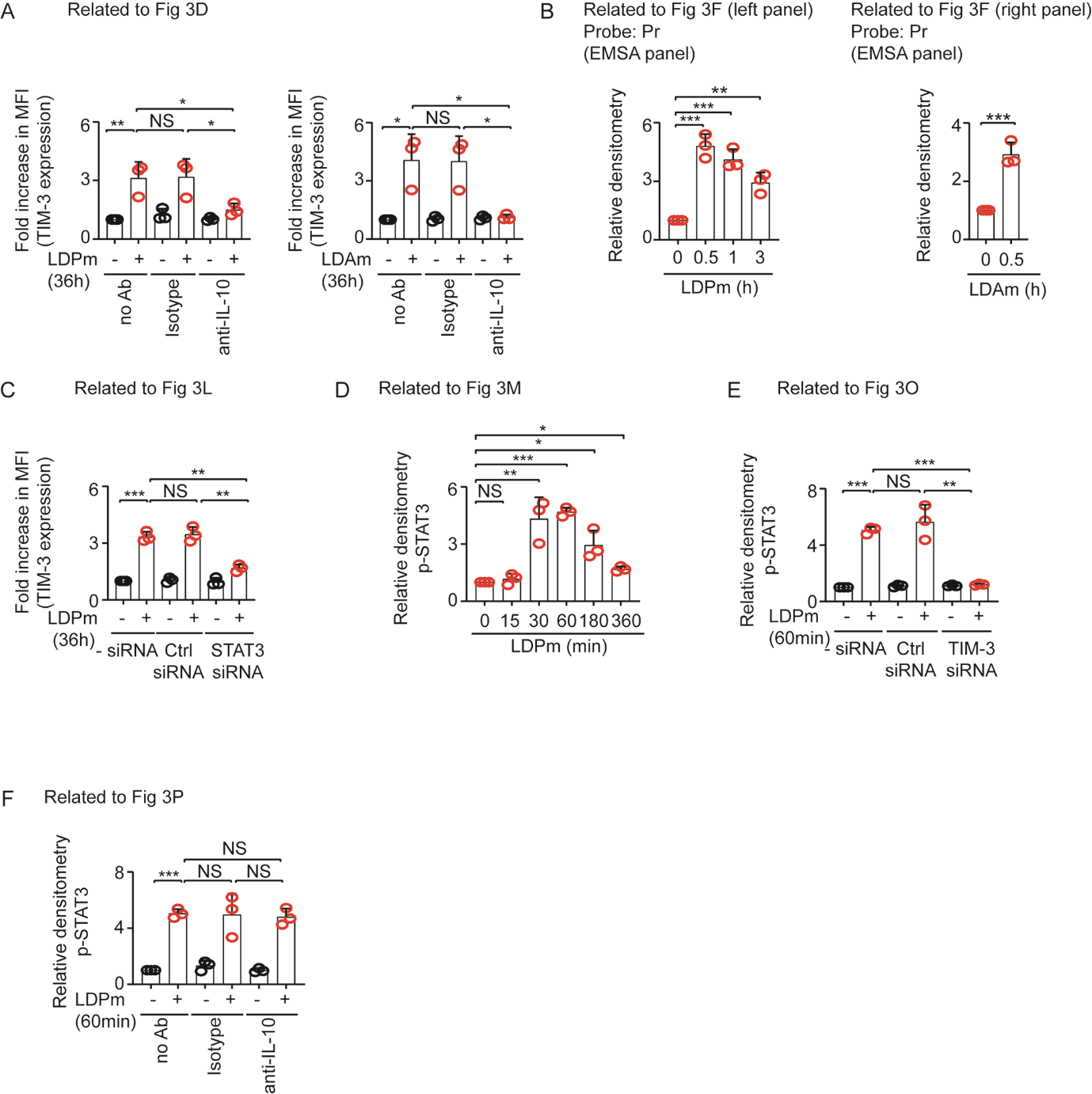
**

**Supplementary Fig. 4 Supporting information for Fig. 3.** **A** Related to Fig. 3D. The flow cytometry data for TIM-3 expression on BMDCs left uninfected (-) or infected (+) with LDPm (left panel) or LDAm (right panel) for 36 h in the presence of indicated antibodies have been shown in Fig. 3D. Corresponding MFIs of TIM-3 expression was determined as described in Supplementary Fig. 3 and presented as fold change relative to uninfected BMDCs cultured with no antibody (no Ab). The combined data of *n* = 3 are presented here. **B** Related to Fig. 3F. Bar graphs showing compiled relative densitometry results (from *n* = 3) of EMSA experiments (shown in Fig. 3F) that were performed using specified probes and the nuclear extracts of BMDCs infected with LDPm (left panel) or LDAm (right panel) for indicated times. Densitometry analysis was performed as described in Fig. 3F. **C** Related to Fig. 3L. BMDCs were transfected with indicated siRNAs and then infected (+) or not (-) with LDPm for 36 h. The representative flow cytometry data for TIM-3 expression has been shown in Fig. 3L. Corresponding MFIs of TIM-3 expression were calculated as in Supplementary Fig. 3 and presented as fold change relative to BMDCs left untransfected (- siRNA) and uninfected (- LDPm). Compiled data of *n* = 3 is presented here as a bar diagram. **D** Related to Fig. 3M. A compilation of relative densitometry results (*n* = 3) for immunoblot analysis (shown in Fig. 3M) of phosphorylated STAT3 in BMDCs infected with LDPm for indicated times has been presented here as a bar graph. The densitometry analysis was performed as described in Fig. 3M. **E** Related to Fig. 3O. Bar graph depicts the relative densitometry results (compiled data of *n* = 3) for immunoblot analysis (shown in Fig. 3O) of phosphorylated STAT3 in BMDCs transfected with indicated siRNAs and then infected with LDPm for 60 min (+) or left uninfected (-). Densitometry analysis was performed as described in Fig. 3O. **F** Related to Fig. 3P. Graphical presentation of relative densitometry results (data combined from *n* = 3 replicates) for immunoblot analysis (depicted in Fig. 3P) of phosphorylated STAT3 in lysates of BMDCs infected (or not) for 60 min with LDPm in the presence of indicated antibodies. The densitometry analysis was performed as described in Fig. 3P. For all panels, each symbol denotes data of an individual replicate. Error bars represent SD. ^*^*p* < 0.05, ^**^*p* < 0.01, ^***^*p* < 0.001; NS, not significant.

**

**

**Supplementary Fig. 5 STAT3-binding site contributes to the *IL-10* promoter activity. A** Schematic of the mouse *IL-10* promoter-firefly luciferase (Luc) constructs used for reporter assay. The expression of firefly luciferase is driven by the mouse *IL-10* promoter fragment (-1541 to -1) containing either wild-type [*IL-10* (Wt) pro] or mutated STAT3 binding site [*IL-10* (Mut) pro]. **B** Activity of the wild-type or STAT3 site-mutated *IL-10* promoter (illustrated in **A**) was measured at 24 h after transfection of HEK293T cells with control vector (Ctrl vec; pBabe) or STAT3-expressing vector; results were normalized to the activity of renilla luciferase (internal control) and presented relative to the cells co-transfected with wild-type *IL-10* promoter-luciferase reporter construct and control vector (pBabe). The compiled data of *n* = 6 is presented here. Each symbol represents data of an individual replicate. Error bars represent SD. ^***^*p* < 0.001.


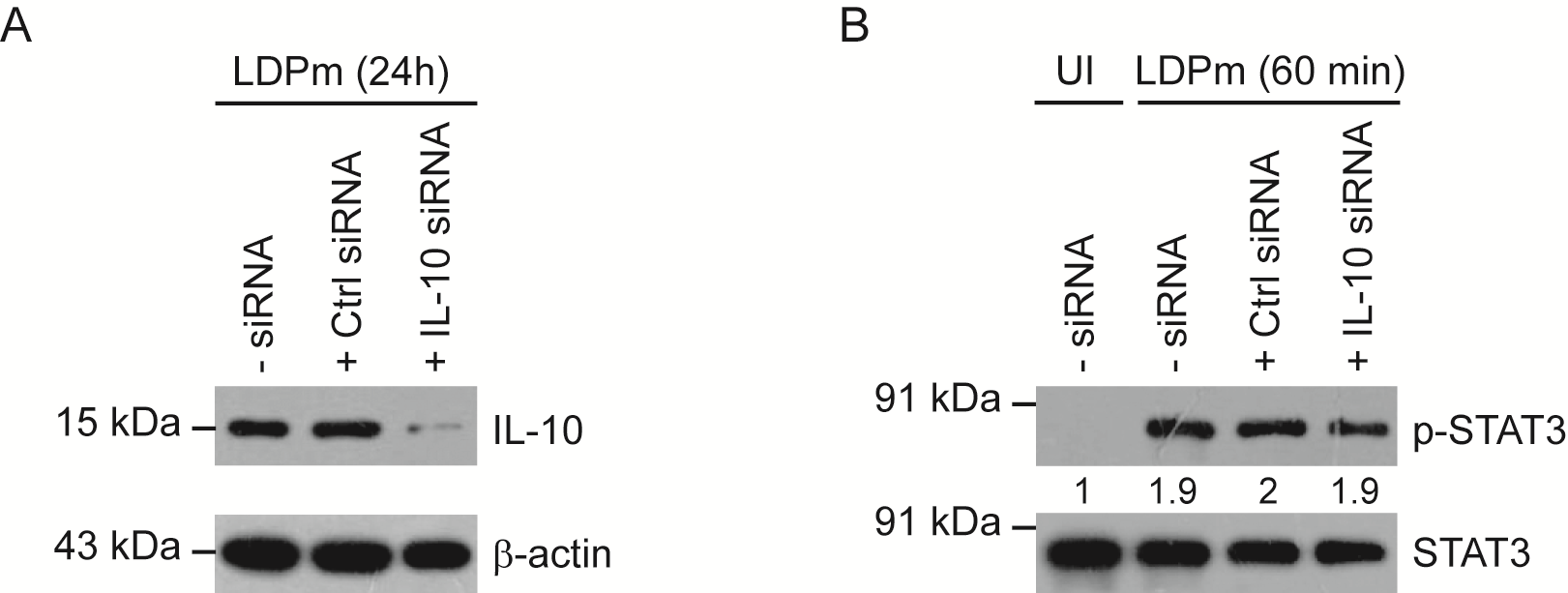


**Supplementary Fig. 6 Silencing of IL-10 does not affect LD-induced STAT3 activation in DCs. A** Immunoblot analysis to detect the expression of IL-10 and β-actin in BMDCs left untransfected or transfected with control siRNA or IL-10-specific siRNA and then infected with LDPm for 24 h, followed by brefeldin A (5 μg/ml) treatment for last 5 h (during 24 h infection time). **B** Assessing (by immunoblot analysis) the effect of IL-10 silencing on the expression of phosphorylated STAT3 in BMDCs infected with LDPm for 60 min. Numbers below lanes represent relative densitometry as in Fig. 3M. Data are representative of two different experiments.


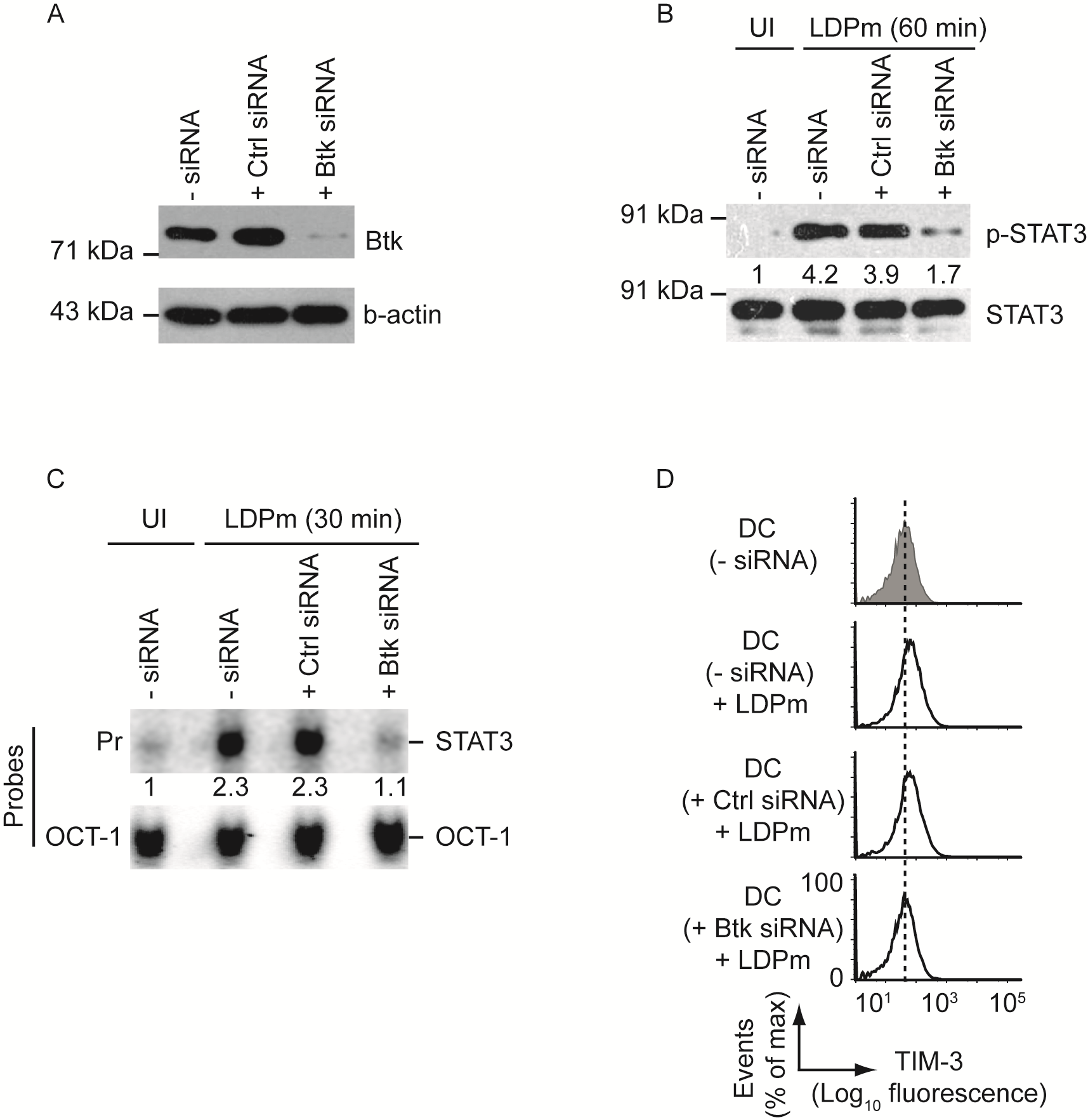


**Supplementary Fig. 7 Btk is required for LD-induced STAT3 activation, binding of STAT3 to the *IL-10* promoter, and TIM-3 upregulation on DCs. A** Immunoblot analysis depicting the efficiency of siRNA-mediated suppression of Btk expression in DCs. Expression of β-actin serves as a loading control. **B** Immunoblot analysis examining total and phosphorylated STAT3 expression in LDPm-infected BMDCs (infection was done for 60 min) after Btk silencing by siRNA. Numbers below lanes represent relative densitometry; measured as in Fig. 3M. **C** EMSA of nuclear extracts of BMDCs transfected with indicated siRNAs and then infected with LDPm for 30 min; assessed with probes as in Fig. 3F. Numbers below lanes indicate relative densitometry; measured as described in Fig. 3F. **D** Flow cytometry analysis of TIM-3 expression on BMDCs transfected with specified siRNAs (left margin) and infected with LDPm for 36 h. UI, uninfected BMDCs. Data are representative of two different experiments.


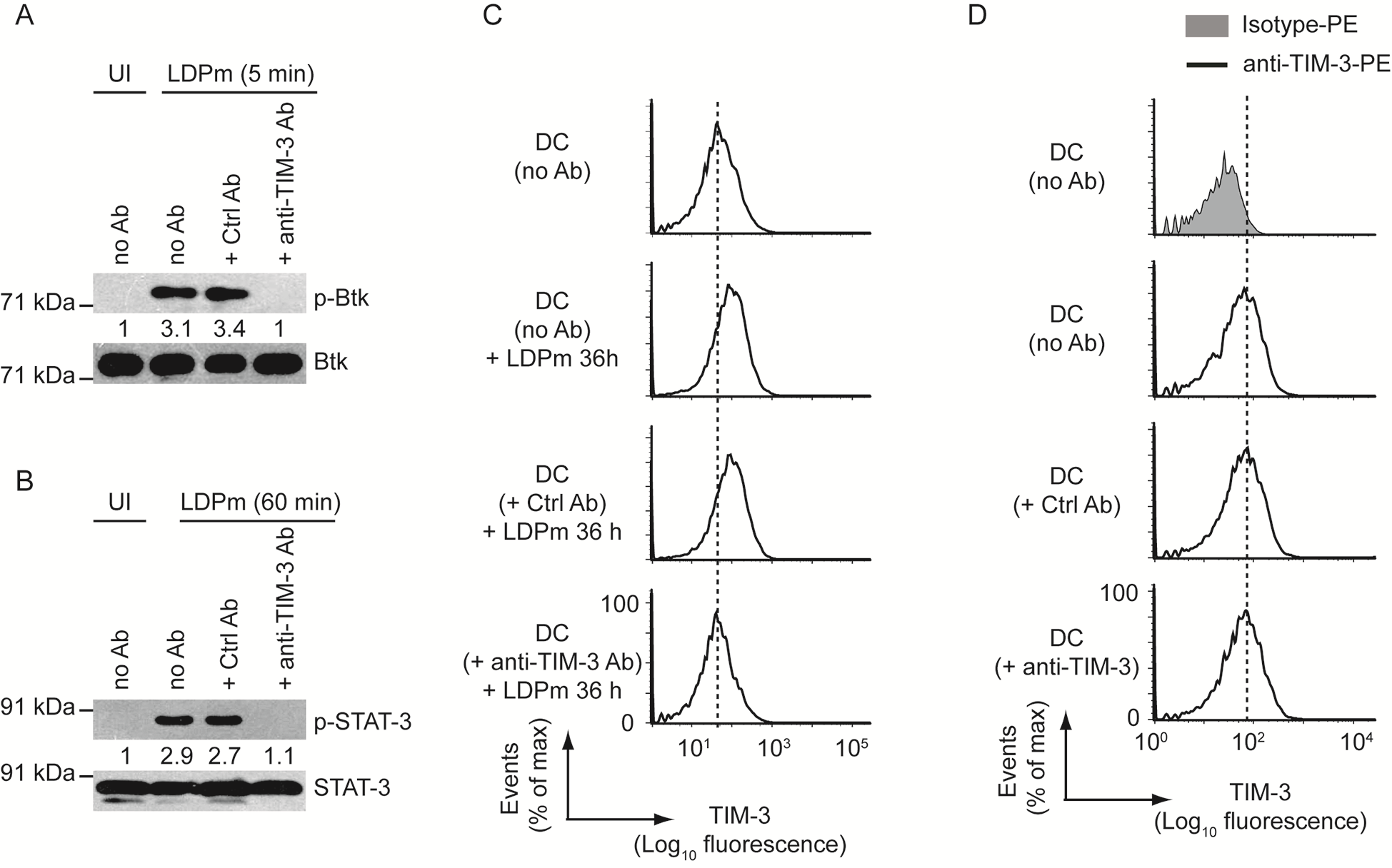


**Supplementary Fig. 8 Blockade of TIM-3 with anti-TIM-3 antibody prevents LD-induced Btk and STAT3 phosphorylation, and TIM-3 upregulation on DCs. A, B** Immunoblot analysis examining the effect of TIM-3 blockade with anti-TIM-3 antibody on the expression of phosphorylated Btk (**A**) or phosphorylated STAT3 (**B**) in BMDCs infected with LDPm for 5 min (**A**) or 60 min (**B**). The expression of total Btk (**A**) or STAT3 (**B**) serves as a loading control. Numbers below lanes indicate densitometry of phosphorylated Btk (**A**) or phosphorylated STAT3 (**B**), normalized to Btk (**A**) or STAT3 (**B**) and presented relative to uninfected BMDCs given no antibody (no Ab) treatment. **C** Analysis of TIM-3 expression on BMDCs infected with LDPm for 36 h after anti-TIM-3 antibody treatment; assessed by flow cytometry. **D** The surface expression of TIM-3 on BMDCs left untreated (no Ab) or treated for 1 h with isotype control antibody or anti-TIM-3 antibody; analyzed by flow cytometry. Data are representative of two different experiments.

**
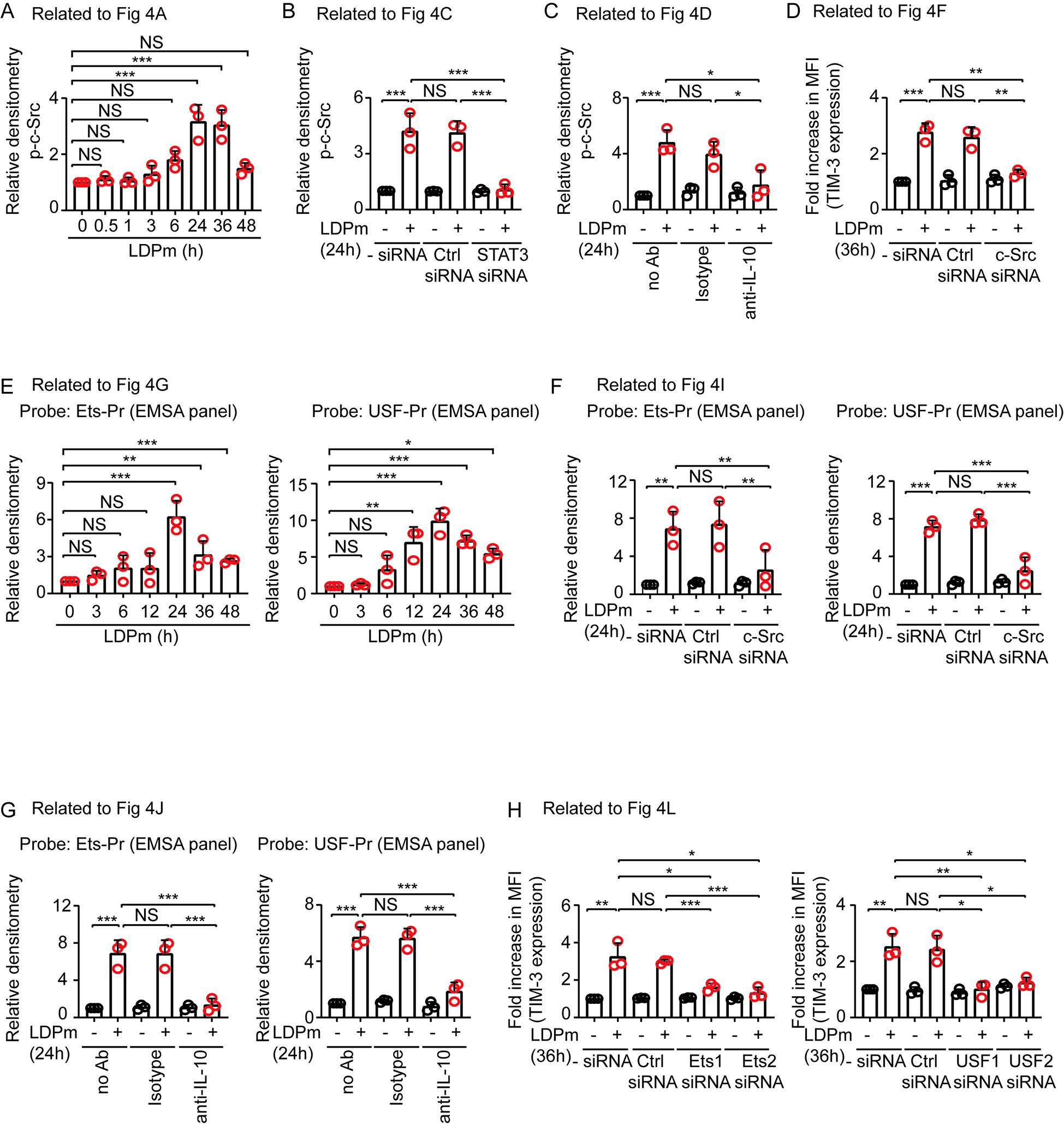
**

**Supplementary Fig. 9 Supporting information for Fig. 4. A** Related to Fig. 4A. Immunoblot analysis of phosphorylated c-Src in BMDCs infected with LDPm for various times has been shown in Fig 4A. Corresponding relative densitometry results (pooled data from *n* = 3) are plotted here graphically. Densitometry analysis was performed as in Fig. 4A. **B** Related to Fig. 4C. Bar graph showing the relative densitometry results (pooled data of *n* = 3) for immunoblot analysis (presented in Fig. 4C) of phosphorylated c-Src in BMDCs transfected with indicated siRNAs and then infected for 24 h with LDPm (+) or left uninfected (-). Densitometry analysis was carried out as in Fig. 4C. **C** Related to Fig. 4D. Relative densitometry results (data compiled from *n* = 3) for immunoblot analysis (shown in Fig. 4D) of phosphorylated c-Src in BMDCs left uninfected or infected with LDPm for 24 h in the presence of indicated antibodies are presented as a bar graph. **D** Related to Fig. 4F. The bar graph shows MFI values of TIM-3 expression on BMDCs transfected with specified siRNAs and then infected with LDPm for 36 h or left uninfected (representative flow cytometry data has been shown in Fig. 4F). The MFI of TIM-3 expression was calculated as in Supplementary Fig. 3 and presented as fold change relative to BMDCs left untransfected (- siRNA) and uninfected (- LDPm). The compiled data of *n* = 3 is presented here. **E** Related to Fig. 4G. Pooled data (from *n* = 3) for densitometry results (relative) of EMSA (presented in Fig. 4G) showing the binding of nuclear Ets and USF transcription factors derived from BMDCs infected with LDPm (for various times) to indicated probes have been presented as bar graphs. Densitometry analysis was done as in Fig. 4G. **F** Related to Fig. 4I. Graphs show relative densitometry results (data compiled from *n* = 3) for EMSA (Fig. 4I) that had been performed using nuclear extracts of BMDCs transfected with indicated siRNAs and infected (or not) with LDPm for 24 h to assess the binding of nuclear Ets and USF transcription factors to Ets-Pr and USF-Pr probes, respectively. Densitometry analysis was performed as in Fig. 4I. **G** Related to Fig. 4J. Bar graphs depicting compiled (*n* = 3) relative densitometry data for EMSA (shown in Fig. 4J) examining the binding of Ets and USF to Ets-Pr and USF-Pr probes, respectively; assessed using nuclear extracts of BMDCs infected (or not) with LDPm for 24 h in the presence of indicated antibodies. Densitometry analysis was carried out as in Fig. 4J. Each symbol represents data of an individual replicate. **H** Related to Fig. 4L. The bar graphs depict MFI values of TIM-3 expression on BMDCs transfected with indicated siRNAs and then left uninfected or infected for 36 h with LDPm (representative flow cytometry data has been shown in Fig. 4L). The MFI of TIM-3 expression was calculated as in Supplementary Fig. 3 and presented as fold change relative to BMDCs left untransfected (- siRNA) and uninfected (- LDPm). The compiled data of *n* = 3 is presented here. Error bars represent SD. ^*^*p* < 0.05, ^**^*p* < 0.01, ^***^*p* < 0.001; NS, not significant.

**
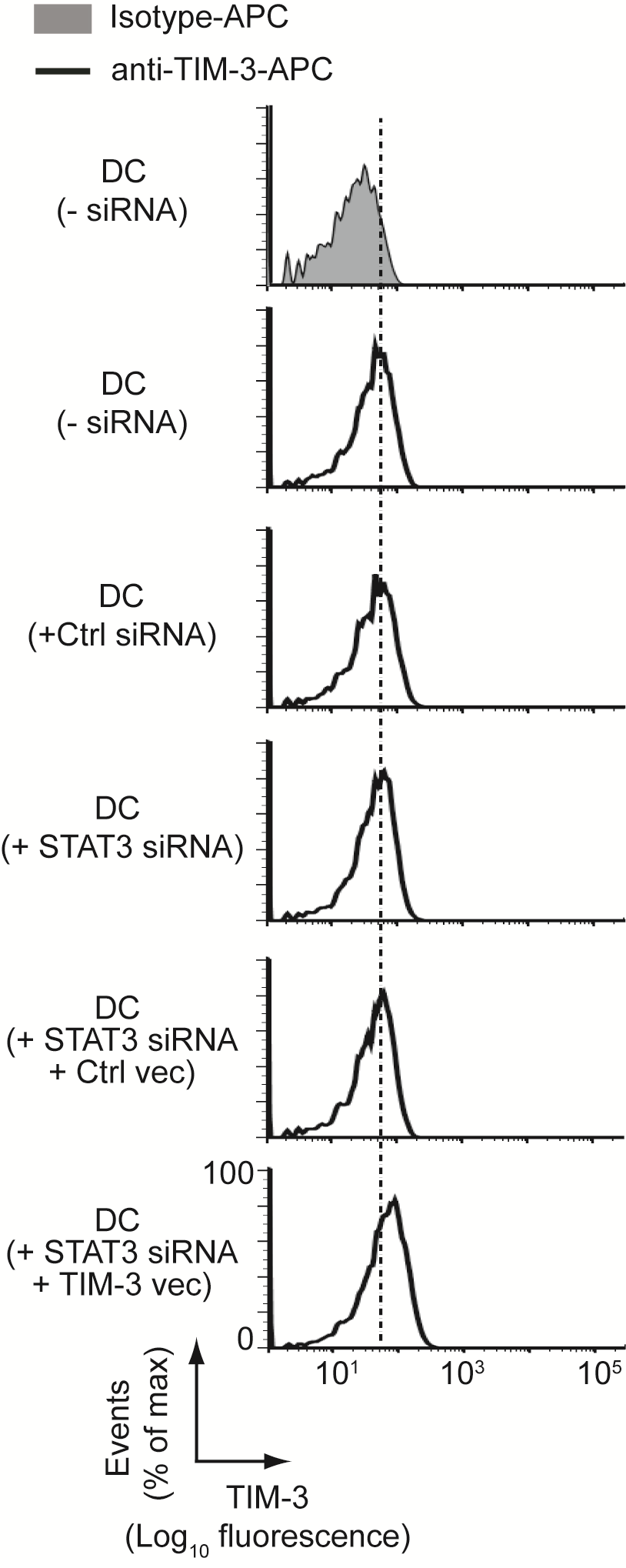
**

**Supplementary Fig. 10 Analysis of TIM-3 expression on STAT3-silenced DCs after TIM-3 overexpression.** BMDCs were transfected (or not transfected) with control (Ctrl) siRNA or with STAT3-specific siRNA either alone or together with the control vector (Ctrl vec) or TIM-3-expressing vector (TIM-3 vec). The expression of TIM-3 on BMDCs was evaluated via flow cytometry (representative data out of *n* = 2).

**
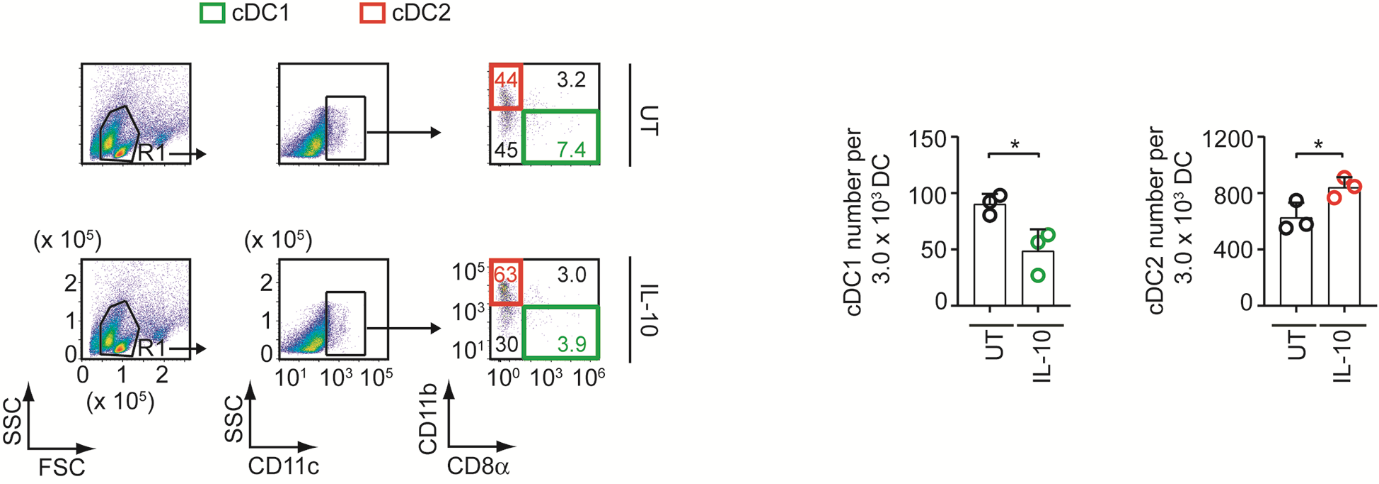
**

**Supplementary Fig. 11 IL-10 increases cDC2 abundance.** The frequency (left) and number [per 3 x 10^3^ DCs (i.e., CD11c^+^ gated cells); right] of cDC1 and cDC2 population following *in vitro* treatment of splenocytes (from uninfected mice) with IL-10 for 72 h; assessed by flow cytometry (representative data of *n* = 3; left). Right, bar graphs show compiled data (*n* = 3). Each symbol represents data of an individual replicate. Error bars indicate SD. ^*^*p* < 0.05. UT, untreated splenocytes.
